# A comprehensive review of advanced trends: from artificial synapses to neuromorphic systems with consideration of non-ideal effects

**DOI:** 10.3389/fnins.2024.1279708

**Published:** 2024-04-10

**Authors:** Kyuree Kim, Min Suk Song, Hwiho Hwang, Sungmin Hwang, Hyungjin Kim

**Affiliations:** ^1^Department of Electrical and Computer Engineering, Inha University, Incheon, Republic of Korea; ^2^Division of Nanoscale Semiconductor Engineering, Hanyang University, Seoul, Republic of Korea; ^3^Division of Materials Science and Engineering, Hanyang University, Seoul, Republic of Korea; ^4^Department of AI Semiconductor Engineering, Korea University, Sejong, Republic of Korea

**Keywords:** artificial intelligence, neural network, synaptic device, neuromorphic system, non-volatile memory, in-memory computing, hardware non-idealities, array operation

## Abstract

A neuromorphic system is composed of hardware-based artificial neurons and synaptic devices, designed to improve the efficiency of neural computations inspired by energy-efficient and parallel operations of the biological nervous system. A synaptic device-based array can compute vector–matrix multiplication (VMM) with given input voltage signals, as a non-volatile memory device stores the weight information of the neural network in the form of conductance or capacitance. However, unlike software-based neural networks, the neuromorphic system unavoidably exhibits non-ideal characteristics that can have an adverse impact on overall system performance. In this study, the characteristics required for synaptic devices and their importance are discussed, depending on the targeted application. We categorize synaptic devices into two types: conductance-based and capacitance-based, and thoroughly explore the operations and characteristics of each device. The array structure according to the device structure and the VMM operation mechanism of each structure are analyzed, including recent advances in array-level implementation of synaptic devices. Furthermore, we reviewed studies to minimize the effect of hardware non-idealities, which degrades the performance of hardware neural networks. These studies introduce techniques in hardware and signal engineering, as well as software-hardware co-optimization, to address these non-idealities through compensation approaches.

## 1 Introduction

The neuromorphic system, designed to mimic the neuron-synapse connections of the human neural network, aims to achieve a robust and efficient operation in big data-based deep learning and artificial intelligence (AI) systems (Mead, 1990; Ham et al., 2021). To accomplish this goal, it focuses on key characteristics of biological neural networks, such as large-scale parallel signal processing and ultra-low power consumption. Over the past few decades, extensive research has been conducted on artificial neural network (ANN) algorithms, which emulate biological neural networks using a mathematical perceptron (Jain et al., 1996; Maass, 1997; Gardner and Dorling, 1998). These ANN algorithms include various network structures like convolutional neural networks (CNNs), used for image classification through kernel-based feature extraction, and fully connected networks (FCNs) with multiple perceptron layers (Albawi et al., 2017; Gu et al., 2018). In general, ANNs operate in two phases: the ‘training’ phase, where interconnected synaptic weights are adjusted in the direction of the desired output based on gradient descent with respect to the loss function, and the ‘inference’ phase, where output values are determined through the vector–matrix multiplication (VMM) of input data and weights. Throughout this phase, neuron outputs are represented using activation functions such as sigmoid, ReLU, tanh, and others.

**Figure 1:**
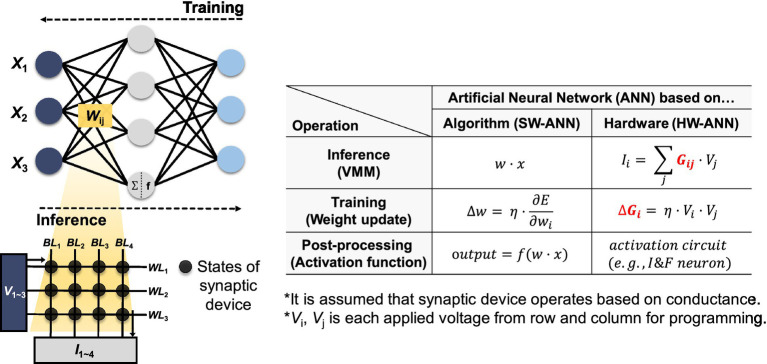
Correspondence between SW-ANN and HW-ANN realized by synaptic device array and the correlation between SW-ANN and HW-ANN. Red text indicates that it is implemented by synaptic devices.

Neuromorphic systems can perform powerful and efficient neural computations by hardware implementations of ANNs (Misra and Saha, 2010; Indiveri and Liu, 2015; Schuman et al., 2017), and the basic concept of hardware-based neural networks (HW-ANNs) with synaptic devices is described in Figure 1. In HW-ANNs, the two phases of the ANN algorithm can be realized by reading and adjusting the states of synaptic devices for inference and training, respectively. The SW-ANN model can be implemented as array structure-based hardware consisting of several synaptic elements. The ANN model has three core components: input vector *x*, weight matrix *w*, and output vector *f*(*w*·*x*), where *f* is the activation function. When these components are implemented in hardware, they have the following correspondence: (1) Input vector *x* corresponds to signals applied to the wordlines (WLs) of the synapse device array. For example, in the case of the Modified National Institute of Standards and Technology database (MNIST), images are composed of 28 × 28 pixels with 8 bits per pixel. Such images are encoded into waveforms based on pulse amplitude or repetition count and then presented as input to the neural network. (2) The weight matrix, denoted as *w*, is implemented by the state of synapse devices. Assuming a conductance-based device, it is symbolized as *G*. (3) The output vector, denoted as *f*(*w*·*x*), which serves as the input vector to the next layer, corresponds to the output of the CMOS peripheral circuit. The output current (*I*_i_) at each BL can be determined by the VMM operation of the input voltage signals (*V*_j_) applied to the WLs and the device state. Neuron circuits in HW-ANNs correspond to activation functions in SW-ANNs and are implemented in analog or mixed-signal circuits to represent the output signal generation or firing of neurons. These circuits receive presynaptic signals from synaptic devices, and then generate corresponding output signals when integrated signals exceed a certain threshold.

Generally, incorporating synaptic devices into large-scale neuromorphic systems demands consideration of specific performance metrics aligned with industrial requirements. These metrics include (1) non-volatile memory, which is essential for maintaining network function by reliably storing weight values; (2) high integration density is necessary to efficiently accommodate a large neural network in a limited area; (3) high operating speed is needed for fast inference (read) and training (write) operations in HW-ANNs; (4) low power consumption is desired to achieve energy-efficient neural network operations, including both inference and training. Various types of synaptic devices have been investigated for these purposes and can be categorized into two types: conductance-based and capacitance-based. Among conductance-based devices, there are two-terminal devices exhibiting memristive behaviors (Strukov et al., 2008) and three-terminal devices with an additional selecting electrode such as a gate for the channel conductivity. Synaptic array architectures, which consist of synaptic device cells, can vary based on the device operation mechanism and structure, each having its unique VMM operations depending on the array structure. Furthermore, the neuromorphic system can be affected by the inherent non-ideal characteristics of synaptic devices. Due to material and process-related limitations, synaptic devices may exhibit non-linear and asymmetric conductance modulations, a narrow on/off ratio, low precision of device states, poor reliability, and device-to-device or cycle-to-cycle variations. These challenges make it difficult to achieve reliable and accurate analog computing operations, including computations with analog states, compared to conventional digital computing. To mitigate this issue, device-to-circuit- and circuit-to-algorithm-level studies have been conducted to explore compensation methods for addressing hardware non-idealities.

This article provides a comprehensive investigation into neuromorphic systems with non-volatile memory devices. This article explores the significance of synaptic device metrics in various applications and discusses their importance. Firstly, synaptic devices are discussed depending on the operation mechanism and device structure in Section 2. Furthermore, the corresponding array structure is explored, including weight mapping schemes with recently reported advances, such as capacitor-based synapses. In Section 3, we have introduced studies that explored the compensation methods to mitigate hardware non-idealities from two perspectives. Given inevitable non-idealities, compensation approaches against the non-idealities are discussed, including hardware and signal engineering as well as software-hardware co-optimization methods to specifically address non-idealities and enhance neuromorphic system performance. We believe that this review article could contribute to a better understanding of recent advances in neuromorphic system engineering and the development of hardware-driven neural network systems, even in the presence of non-idealities.

## 2 Synaptic devices

**Figure 2:**
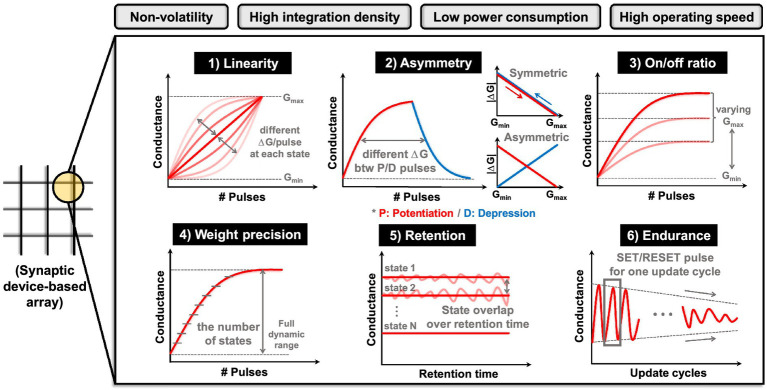
Requirements for large-scale neuromorphic systems and each performance metric for synaptic device.

The essence of a neuromorphic system resides in synaptic devices that not only fulfill ‘store’ functions but also execute ‘computational’ operations. In other words, synaptic devices must be capable of both storing the states and accurately representing analog values while also performing precise VMM operations. These functionalities hold immense importance as they directly influence the performance of neural network applications. The following are the required performance metrics that synaptic devices should exhibit, as depicted in Figure 2 (Misra and Saha, 2010; Chen et al., 2017; Yu, 2018). (1) Linearity: it indicates how much the conductance of a device changes in relation to the applied pulses. Better linearity means that the amount of weight change is consistent in relation to the number of applied pulses. This factor plays a critical role in weight modulation to reach a target device state. It also affects the precision of weight states, along with the time and energy necessary for weight modulation. Linearity can be analyzed through the conductance response (*G* response) according to the number of pulses. (2) Asymmetry: it refers to the disparity in the amount of conductance change (Δ*G*) when the device undergoes potentiation (increasing conductance) or depression (decreasing conductance). This implies that Δ*G* depends on the current state of the device. Thus, when certain pulses are applied to the current state, an imbalance arises in the conductance increase and decrease, which subsequently affects the precision of weight adjustment. Asymmetry can be determined from *G* response, and *G* versus Δ*G* response data can provide a more intuitive interpretation. (3) On/off ratio: it is also referred to as the dynamic range, the on/off ratio signifies the proportion between the maximum conductance (*G*_max_) and the minimum conductance (*G*_min_) achievable by the device. The higher dynamic ranges provide sufficient margins between weight states, guaranteeing weight precision and establishing a more stable state representation. (4) Precision: it means the number of states that a device can exhibit within its dynamic range, corresponding to the concept of multi-bit characteristics in the conventional memory device. It entails maintaining intervals between device states to achieve multi-bit representation, aligning with the objective of preventing overlaps. The attainable number of states relies on the distribution of states resulting from device variability. Weight precision is closely related to the capability of analog value implementation. (5) Retention: it is a reliability metric for memory devices, indicating their ability to maintain their current weight states effectively. To assess it consistently over an extended period, changes in conductance state are analyzed under high-temperature conditions. (6) Endurance: it is also a reliability metric that quantifies how many switching cycles a memory device can endure. It evaluates the number of times the device can switch during set/reset or program/erase pulse cycles, serving as a measure of the weight update lifetime and directly impacting the overall performance of HW-ANNs.

In addition to these considerations, the switching (write/read) speed of synapse devices, which represents the fundamental level of the overall system, must be carefully addressed to efficiently implement large-scale HW-ANNs. Slow operation of synaptic devices can lead to decreased throughput and increased latency, making it a critical metric for real-time applications across the entire system (Zhang W. et al., 2019). Moreover, power consumption is a crucial requirement at the device level. Energy efficiency, from a device design perspective, is closely tied to the switching operating voltage of memory devices. Balancing power consumption and operational stability requires careful design and configuration of switching/read voltage amplitudes, especially to achieve selective operation on target cells (van De Burgt et al., 2018). However, it is important to note that the metrics associated with synaptic devices, while critical, do not always collectively fulfill ideal characteristics. For instance, achieving excellent retention may come at the cost of low endurance, and weight precision may vary based on factors such as on/off ratio and programming characteristics. Furthermore, consistently defining optimal synapse device characteristics proves challenging due to diverse considerations such as target application, network topology, and system-wide optimization. More details on these challenges are provided below.

**Figure 3:**
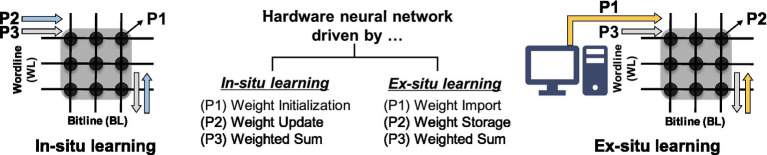
Operating phases in hardware neural networks and required metrics for each phase.

There are two HW-ANN learning methods: *in-situ* (online) learning and *ex-situ* (offline) learning. *In-situ* learning involves conducting training directly on the hardware itself. This method possesses tolerance and self-adapting capabilities toward hardware imperfections (Li et al., 2018). On the other hand, the *ex-situ* approach involves training in software and then importing the pre-trained weights into the neuromorphic system. *Ex-situ* learning benefits from the direct use of training algorithm in software-based artificial intelligence, resulting in higher performance. Figure 3 explains the operation phases of each learning method. The primary action in *in-situ* learning is weight update, while *ex-situ* learning focuses on transferring the pre-trained weights, referred to as weight import. Consequently, the importance of specific metrics in the synaptic devices varies according to the learning method. In other words, the required metrics for synaptic devices are application-dependent.

The requirements of each metric can be prioritized based on the characteristics of the target application. (1) Frequency of state writing/reading: In classification applications (or inference machines, write-once-read-only systems), the stored memory state is read repeatedly, necessitating uniform reading at every step. Therefore, metrics such as retention, read stability, read speed, and a wide on/off ratio are of greater importance. Conversely, for on-chip training applications (such as pulse modulation, online learning for DNN), frequent weight update-state writing is required in hardware. Achieving this necessitates the use of a constant programming pulse, thus requiring synapse devices with robust endurance to sustain operational range during frequent and swift switching. Precise linear and symmetric programming, along with high bit precision, is essential to clearly represent different states (Sun and Yu, 2019; Zhao et al., 2020). (2) Network topology: The size of the network and the defined form of input/weight/output also influence metric requirements. As the network size increases, the array size grows accordingly, and design considerations must extend beyond single-device operation to the array level. With an increase in the number of cells, desired values at the single-cell level may change due to inter-cell influence, necessitating faster switching speeds. Furthermore, depending on whether the input/weight/output form of the neural network is binary or analog, requirements for weight precision vary according to the on/off ratio and programming characteristics. (3) Systematic design interface: Additionally, considering the hierarchy of the overall neuromorphic system, specifications of the signal conversion peripheral circuit connected to the synaptic device (such as DAC/ADC precision, parasitic resistance, etc.) as well as the on/off ratio, weight precision, and speed of the synaptic device should be considered.

The desired metric values are outlined in Table 1, where their significance varies depending on the target application, often necessitating trade-offs. Key metrics for enhancing synaptic device characteristics include tuning accuracy during weight import, conductance response for weight updating, long retention time for weight storage, and evaluating the difference in VMM current during weighted sum operations (VMM operations). The importance of each metric can fluctuate depending on specific operational requirements, such as read speed for inference and write speed for frequent weight updates. We will explore the operational principles and metrics of conductance-based devices (both two- and three-terminal devices) as well as capacitance-based devices to achieve these desired synaptic device characteristics.

**Table 1:** Desired figure of merit as synaptic devices and significance of each metric according to operating phase in a hardware-based neural network.

Figure of merit	Desired value	Importance of each metric according to operating phase (larger number, higher requirement)			
		Weight import	Weight update	Weight storage	Weighted sum
Weight programming	Linear and symmetric (small non-linearity; 0.5 ~ 1)^a^	5	5	1	2
On/off ratio	50^b^	4	5	1	5
Weight precision	16 (4 bits)^c^ ~ 64 (6 bits)^b^	5	5	1	2
Retention	10^3^ ~ 10^8^ s, 10 years at 85°C^d^	5	2	4	5
Endurance	~10^9^ cycles (online learning)	2	5	4	2
Write/read speed^e^	<1 μs				

^a^The given numbers are defined by the non-linearity formula, as detailed in Yu et al. (2016). As a result of evaluation with NeuroSIM+ for both online and offline learning modes, non-linearity of 1 or more (high non-linearity) was defined as a value with low resilience against performance degradation due to device-to-device variation. ^b^These values are the ideal characteristics of synaptic device confirmed with NeuroSIM+ (simulation framework for benchmarking HW-ANN). This required value-based HW-ANN was performed on online learning (derived accuracy: 94.8%) and offline classification (accuracy: 94.5%) on the two-layer multilayer perceptron (MLP) neural network with the MNIST dataset (Yu et al., 2016). ^c^Accuracy degraded gradually when the weight precision was lower than 4 bits for ex-situ training. ^d^This figure is widely applied as an industry standard in existing digital computing (memory-centric) and is applicable for the synaptic devices with binary states. ^e^ Speed requirements are different for each target application. There will be stricter metrics for read speed for ex-situ learning (write-once-read-only) and write speed for in-situ learning (frequent weight update).

### 2.1 Two-terminal devices

#### 2.1.1 Resistive random access memory (RRAM)

**Figure 4:**
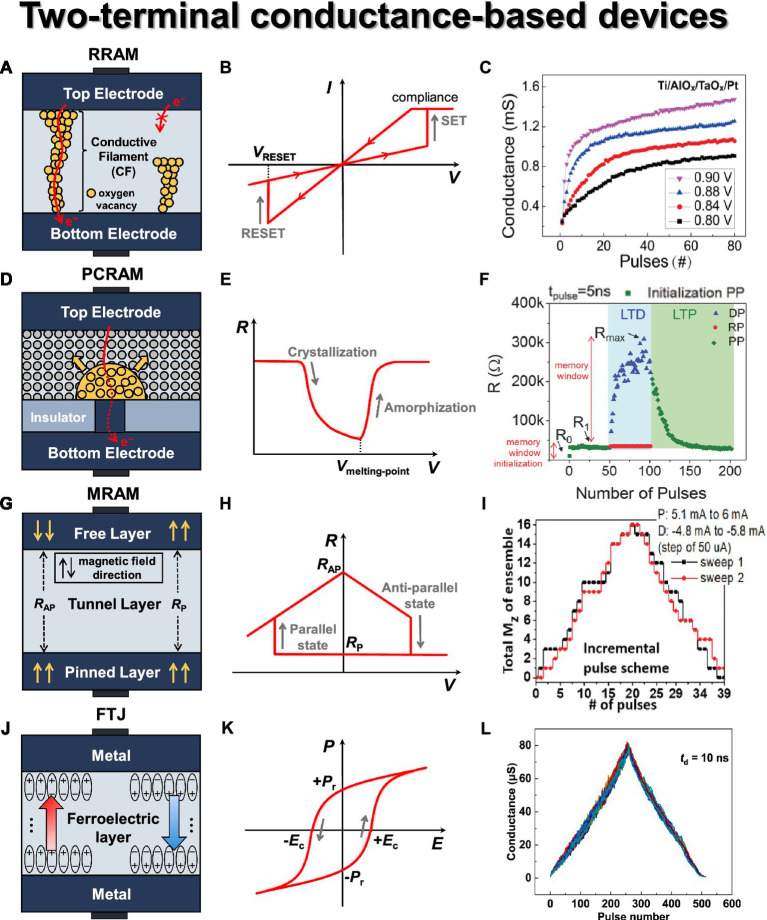
Two terminal conductance-based synaptic devices. **(A)** RRAM basic structure. **(B)** RRAM *I*-*V* curve. **(C)** Conductance modulation on AlO_x_/TaO_x_-based RRAM under identical pulses with different amplitudes. Reproduced with permission (Sun et al., 2018), Copyright 2018 IEEE. **(D)** PCRAM basic structure. **(E)** PCM *R*-*V* curve. **(F)** Conductance modulation on narrow heater electrode-based PCM with identical fast programming pulses of depression, potentiation, and read, resulting in gradual amorphization and crystallization (La Barbera et al., 2018), Copyright 2018 John Wiley & Sons. **(G)** MRAM basic structure. **(H)** MRAM *R*-*V* house curve. **(I)** Conductance modulation on CoFeB/MgO-based MRAM under incremental pulse scheme. Reproduced under the terms of the CC-BY Creative Commons Attribution 4.0 International License (Ostwal et al., 2019), Copyright 2019 IEEE. **(J)** FTJ basic structure. **(K)** FTJ *P*-*E* hysteresis curve. **(L)** Symmetric conductance modulation on FTJ. Reproduced under the terms of the CC-BY Creative Commons Attribution 4.0 International License (Luo et al., 2022), Copyright 2022 Springer Nature.

Resistive random-access memory (RRAM) is one of the most representative two-terminal devices with a metal–insulator–metal (MIM) structure in general, as shown in Figure 4A. Resistance changes occur within an intermediate material known as the switching layer, utilizing a variety of metal oxide materials such as TiO_x_, HfO_x_, AlO_x_, WO_x_, TaO_x_, and others. RRAM can be classified into filamentary type and non-filamentary type (also referred to as interfacial type; Lee et al., 2008; Jang et al., 2015; Wang et al., 2015; Hu et al., 2022). The filamentary type includes OxRAM (metal oxide RRAM) and CBRAM (conductive bridge RAM). OxRAM utilizes metal oxide materials as the switching layer, and oxygen ions migrate toward the top electrode when switching voltage is applied, leading to the formation of conductive filaments (CFs) consisting of oxygen vacancies within the switching layer. Various metal oxide materials can be employed, and in certain cases, a thin bilayer can be introduced to facilitate the development of multiple weaker filaments, enabling gradual switching operations (Gao et al., 2017). CBRAM forms a CF consisting of metal ions and employs materials such as metal oxides, amorphous silicon, and solid electrolytes as the switching layer. Due to its operation mechanism, it is believed to have the potential to be scaled down to an atomic-level dimension. This type of RRAM device exhibits stochastic and abrupt switching characteristics due to the randomness of CF generation. Consequently, this leads to challenges in achieving the desired state because of non-linearity in the conductance response and variations in device reliability caused by fluctuations in the device state. In addition, CBRAM tends to have a relatively high LRS (low resistance state) current due to the metal path within the CF, resulting in increased leakage current. In contrast, the switching materials employed in non-filamentary type devices are not as widespread as those used in filamentary types, leading to increased costs, reduced retention, and slower switching speeds (Gao et al., 2016).

The operation of RRAM can be divided into three phases—forming, set, and reset—which are determined by the states of CFs within the switching layer. Initially, RRAM is in the pristine state, where little to no conductive filament is present in the switching region, resulting in minimal current flow. When voltage is applied to the top electrode, oxygen ions (O^2−^) migrate toward the top electrode, generating oxygen vacancies in the switching region. This results in the formation of CF through which electrons can flow, a process known as the forming phase. During the forming phase, RRAM transitions to the LRS, allowing current to flow easily through the CF. In the LRS, applying *V*_RESET_ to the top electrode induces recombination of oxygen ions near the top electrode, filling the oxygen vacancies and leading to the rupture of the CF. As a result, the device switches to the HRS (high resistance state) with reduced current flow. This process is called the reset operation. In the HRS, applying *V*_SET_ to recreate the conductive filament is known as the SET phase. The current–voltage (*I*-*V*) curve of a typical RRAM device, exhibiting its representative switching behavior, is shown in Figure 4B. Based on this characteristic, it is necessary to determine an appropriate read voltage (*V*_read_) that does not alter the device state.

RRAM offers several advantages, including BEOL (back-end-of-line) compatibility, scalability (4*F*^2^/*n*, where *n* refers to the number of layers in the 3-D structure), nanosecond-level read/write speed, 10^6^ endurance cycles, and low power consumption (Wong et al., 2012). However, RRAM exhibits stochastic resistance states resulting from the randomness of oxygen vacancy generation by oxygen ion trapping and de-trapping. Thus, this leads to device-to-device and cycle-to-cycle variations in conductance values rather than maintaining a stable conductance level Liao et al. (2020) incorporated the number of conductive filaments (*N*), filament gaps (*T*_gap_), and applied voltage (*V*) into the *I*-*V* characteristic of the switching region of multiple-weak-filament-based analog RRAM. This enables us to anticipate the dynamic switching behavior of RRAM devices with stochastic characteristics. Furthermore, based on the mathematical modeling, it provides a reasonable explanation for the weight update behavior within the array structure, and the consistency with experimental results validates the soundness of this model. Kang et al. (2022) proposed a novel cluster-type CBRAM to tackle the challenge of achieving analog resistance changes caused by the electric field feedback effect in conventional filament-type CBRAM. The proposed solution involves precise control over the amount of metal ions in the cluster type. By utilizing a reducing agent, Ti, with a lower standard reduction potential than Ag, the oxidation and reduction processes of Ag cations are carefully regulated. This innovative approach mitigates the electric field feedback effect, resulting in a balance between linear switching characteristics and a high on/off ratio, demonstrating its potential as an exceptional analog synaptic device.

Efforts to achieve gradual switching characteristics in RRAM have primarily focused on the utilization of additive layers such as bilayers (commonly referred to as multilayers; Wang Z. et al., 2018; Kim S. et al., 2022). Sun et al. (2018) fabricated AlO_x_/TaO_x_-based RRAM devices and achieved high uniformity, excellent analog switching characteristics (~200 states), and impressive retention properties (~30,000 s). They conducted a comparison between identical and non-identical pulse inputs to modulate conductance, demonstrating that the modulation of RRAM device weights can be achieved through pulse parameters, as shown in Figure 4C. They observed different conductance responses based on amplitude (*V*_SET_: 0.8–0.9 V) and width (1–15 μs), confirming the potential for weight modulation of RRAM devices. Yeon et al. (2020) fabricated Si-alloy:Ag electrochemical RRAM devices and achieved highly stable operational characteristics. By employing an optimized alloying ratio, they successfully controlled the movement of mobile Ag ions with Cu, resulting in spatial and temporal switching uniformity. They also obtained a conductance range exceeding 10^2^ and significantly enhanced programmed symmetry characteristics.

The fundamental mechanism for the recently reported RTN of RRAM has been demonstrated through conductive atomic force microscopy (C-AFM) and simulations based on the electron wave energy function. RTN arises from electron transport obstruction by trapped charges within incomplete channels, in addition to the main conduction channels. A solution to this issue involves the removal of the RTN component by applying a subthreshold voltage significantly smaller than the set/reset voltage, thereby eliminating incomplete channel islands and phase boundaries (a process known as phase field relaxation). Consequently, achieving high-precision programming is feasible through a stabilization process involving denoising voltage for the RTN components generated within the synapse device. In essence, the development of an RRAM-based neuromorphic chip with remarkable analog functionality, capable of expressing up to 2,048 states through fundamental-level operation analysis from a device perspective, underscores the potential for the commercialization of memristor device-based arrays (Rao et al., 2023).

#### 2.1.2 Phase-change random access memory (PCM)

Phase-change memory (PCM) is a two-terminal device where the resistance state is determined by the phase change of a material located between the two electrodes through the application of heat, as shown in Figure 4D (Raoux et al., 2008; Bruns et al., 2009; Wong et al., 2010). It is simply composed of electrodes, a heater, and phase-change material. Typically, the material of the bottom electrode is deposited and then etched into a trench shape, with its surroundings encapsulated by an insulating material (e.g., SiO_2_). Commonly used phase-change materials include chalcogenide-based materials (e.g., Ge_2_Sb_2_Te_5_ or GST). GST can readily switch between amorphous and crystalline states bidirectionally and can maintain each state for an extended period. When the phase-change material is in the crystalline state, it exhibits LRS; when it is in the amorphous state, on the other hand, it blocks the conduction path between the two electrodes, as depicted in Figure 4D as a dot-dash line, resulting in HRS. When a specific voltage is applied between the top and bottom electrodes, the current flows through the GST material, heating and changing its phase. This region where the change occurs is referred to as the programming region or active region, and it typically exhibits a mushroom-shaped profile due to the current crowding effect.

PCM starts in its initial state (as-fabricated device) with a low-resistive crystalline phase. When relatively small-amplitude voltage pulses are applied over an extended period, the GST material undergoes crystallization due to Joule heating. This heating aligns the atomic arrangements within the material without reaching its melting point. It causes the device state to transition from HRS to LRS, a process known as set (see Figure 4E). On the contrary, when relatively large-amplitude but short-duration voltage pulses are applied, a portion of the GST material undergoes local melting (the melt-quench process), resulting in consequent amorphization and a transition to HRS, a process known as reset. For a read operation, the current state of PCM is sensed by applying a weak electrical pulse that does not induce a significant phase change. PCM has several advantages, including fast SET speed, scalability, high endurance, long data retention over 10 years, and a high dynamic range around 10^3^ (Burr et al., 2016). However, PCM exhibits abrupt RESET characteristics due to the crystallization/amorphization of the phase-change material, as reported by Burr et al. (2015). This implies that the conductance modulation is not consistent with the number of pulses applied. Additionally, a prominent issue in PCM is ‘resistance drift’, where the resistance value increases over time.

Kuzum et al. (2012) fabricated a PCM with W/TiN/GST/TiN stack that shows no degradation up to 10^7^ cycles and demonstrates gradual set/reset operations with progressively increasing voltage pulses, enabling analog behavior. For this device, spike-timing-dependent plasticity (STDP) was experimentally verified, indicating the potential for extending to synaptic PCM arrays in terms of nanoscale and energy efficiency. Ding et al. (2019) fabricated a phase-change heterostructure (PCH) device to achieve continuous resistance states. The PCH is a multilayer structure where phase-change materials and confinement nanolayers (TiTe_2_/Sb_2_Te_3_) are alternately deposited. Given that the nanoscale amorphous Sb_2_Te_3_ layers restrict structural relaxation, this device significantly reduced the resistance drift problem compared to traditional GST-based PCMs (Ielmini et al., 2007). As a result, the enhanced weight resolution achieved through stable set/reset operations makes PCH-based devices promising candidates for synaptic applications in neuromorphic systems. La Barbera et al. (2018) demonstrated the potential for synaptic devices using a narrower bottom electrode-based PCM device. With a memory initialization step before applying switching pulses, they employed identical pulses (*V*_SET_: 1.25 V, *V*_RESET_: 1.6 V) for 50 cycles each, resulting in the switching characteristics shown in Figure 4F. These conditions allowed for conductance modulation to initiate without fully covering the amorphous region above the heater, leading to a more gradual switching behavior. The device exhibited endurance of at least 10^6^ cycles across 145 cells.

#### 2.1.3 Magneto-resistive random access memory (MRAM)

Magneto-resistive RAM (MRAM) is a two-terminal device featuring two ferromagnetic layers separated by a non-magnetic layer (Akerman, 2005; Bhatti et al., 2017). The upper ferromagnetic layer is referred to as the free layer, while the lower ferromagnetic layer is known as the pinned or fixed layer. A thin insulating tunnel barrier is placed between these two layers, as shown in Figure 4G. To change the magnetization direction within the magnetic layers, the current direction in the word line (WL) connected to the lower layer is fixed, while the current direction in the bit line (BL) connected to the upper layer is varied. Through this process, the magnetization direction of the free layer can be freely changed based on the programming voltage. The ferromagnetic layers typically consist of transition metal elements (Fe, Co, Ni, etc.) and their alloys (CoFeB, NiFe, etc.), while the non-magnetic layer mainly consists of insulating materials such as MgO.

The resistance state of MRAM is determined based on the alignment of the magnetic orientations in the two ferromagnetic layers, whether they are parallel or antiparallel. If the magnetic orientations are in the same direction, it results in LRS, also known as the parallel state (*R*_P_). Conversely, if they are in opposite directions, it leads to HRS, or the antiparallel state (*R*_AP_), as depicted in Figure 4H. With increasing integration, interference between adjacent cells has led to read errors and energy inefficiencies, prompting the adoption of spin transfer torque MRAM (STT-MRAM). STT involves directly injecting current into the magnetic layer, where the spin carried by the injected electrons is transferred to the spins in the magnetic layer. This allows for the direct switching of the magnetic orientation. Another type of MRAM that utilizes a different mechanism for magnetization inversion is spin-orbit torque MRAM (SOT-MRAM). It generates spin-orbit effects, including a spin Hall effect within the device that enables the generation of spin currents and changes the magnetic orientation of the free layer to enable switching operations. SOT-MRAM injects current in the horizontal direction, producing more spin current for the magnetic layer. This results in faster operation and lower power consumption compared to STT-MRAM, making it the focus of recent extensive research.

In MRAM, the electron traverse between the two ferromagnetic layers through quantum tunneling in the thin insulating layer. The switching operation is achieved by applying different current directions. When the current flows from the pinned layer to the free layer, the spins of the free electrons align themselves according to the magnetization direction of the pinned layer due to the magnetic exchange coupling energy. This alignment of spin-polarized current exerts a torque on the free layer, causing its magnetization direction to align in parallel with that of the pinned layer. This results in LRS (*R*_P_) during the SET operation, as illustrated in Figure 4H. On the contrary, when the current flows from the free layer to the pinned layer, the free electrons with different spin orientations from the pinned layer exert a torque on the free layer in the opposite direction of its magnetization. This counteracting torque changes the magnetization direction of the free layer, leading to HRS (*R*_AP_) during the RESET operation.

MRAM has several advantages, including high endurance (>10^9^ cycles) and fast switching speed (<100 ns). However, it also has some limitations, such as a limited number of distinct resistance states and a small on/off ratio, which can be drawbacks in its state representation capabilities. The key metric that represents the performance of MRAM is the magnetoresistance (MR) ratio, which indicates the ratio of resistance values between LRS (*R*_P_) and HRS (*R*_AP_), so that MR is defined as (*R*_AP_–*R*_P_)/*R*_P_. To achieve high performance during the inference process, high tunnel magnetoresistance (TMR), low write error rate (WER, <10^−6^), and low read disturbance rate (RDR, <10^−6^) are required during the weight import and weighted sum phases (Xu et al., 2018). Zhang et al. (2021) fabricated a high-TMR perpendicular MTJ device with W inserted into the free layer using the following stack: W/CoFeB/MgO/CoFeB/W/CoFeB/MgO/Ta. The device exhibited a 200% TMR at the nanoscale level, and the strong domain wall pinning effect in the free layer facilitated memristive behavior. The plasticity characteristics (resistance changes over time) for ramped and constant voltage pulse sequences were studied. By applying a subthreshold voltage to the free layer with a positive or negative delay Δ*t* following a pre-spike, the resistance was effectively increased or decreased, simulating the STDP characteristic of a biological synapse.

Siddiqui et al. (2019) demonstrated the fabrication of MTJ devices utilizing the magnetic domain wall effect with CoFeB ferromagnetic layers and MgO spacer, which enabled the generation of linear multilevel weights. Additionally, non-linear activation functions were implemented using MTJ devices with sigmoid-like behavior, indicating the versatility of MTJ devices in neuromorphic systems. The linear resistance (weight) changes were observed in a parallel connection of seven MTJ devices when subjected to positive/negative currents in the CoFeB/Ta wire. Ostwal et al. (2019) fabricated a perpendicular SOT-MRAM device based on a Ta/CoFeB/MgO/Ta stack. This configuration yielded a high TMR and gradual potentiation/depression characteristics, as depicted in Figure 4I. In the case of an identical pulse scheme, they repetitively applied a current pulse of 5.4 mA for potentiation and a current pulse of 5.2 mA for depression. In the case of an incremental pulse scheme, they applied a current pulse ranging from 5 mA to 6 mA for potentiation and pulses ranging from −4.8 mA to −5.8 mA for depression, with increments and decrements of ±0.05 mA. Through this approach, they achieved improved linearity and demonstrated the potential of SOT-MRAM as synaptic devices.

#### 2.1.4 Ferroelectric tunneling junction (FTJ)

Ferroelectric memory can be utilized as both capacitor-based types, such as ferroelectric random-access memory (FeRAM) and ferroelectric capacitor, and conductance-based types, including ferroelectric field-effect transistor (FeFET) and ferroelectric tunneling junction (FTJ; Oh et al., 2019; Slesazeck et al., 2019; Mikolajick et al., 2022). Ferroelectric memory functions by storing information through the polarization of ferroelectric material when an external electric field is applied, as illustrated in Figure 4J. Ferroelectric materials have a polarization-electric field (*P–E*) hysteresis due to their non-centrosymmetric structure, as shown in Figure 4K. Unlike most materials that lose their polarization properties once the electric field is removed, non-centrosymmetric FE materials maintain polarization even without the electric field. The polarization state when the electric field is zero is termed as remnant polarization (*P*_r_), and this lattice structure and the presence of electric dipoles give rise to two distinct polarization states (+*P*_r_ and − *P*_r_). The absence of a central positive charge leads to an electric dipole moment, often resulting in two stable configurations. Altering the state of *P*_r_ involves applying an electric field greater than a threshold value known as the coercive field, which signifies the reverse field necessary to nullify the polarization state.

Among ferroelectric devices, FTJ is a two-terminal device that utilizes ferroelectric materials for practical memory applications (Kohlstedt et al., 2005; Böscke et al., 2011; Garcia and Bibes, 2014). Operating on the principle of electron tunneling, this memory necessitates an exceedingly thin ferroelectric film. Thus, HfO_2_-based material capable of deposition in ultrathin layers has predominantly been used. The FTJ features a metal-ferroelectric-metal sandwich structure and shares similarities in configuration with RRAM. The process of polarization reversal is employed to alter the effective tunneling barrier between the electrodes. Charge carriers accumulate or deplete within the interface layers of electrodes to screen bound polarization charges, depending on the polarization direction within the tunneling barrier. In junctions employing distinct electrodes, this screening effect induces an uneven barrier potential. By using top and bottom electron electrodes with two different screening lengths, the tunneling probability varies depending on the polarization direction. The state is classified based on the difference in tunneling current generated from the read bias, and tunneling electro-resistance (TER) is a method used to control the tunneling current through polarization. TER is measured by calculating the resistance ratio in LRS and HRS (Gruverman et al., 2009; Zhuravlev et al., 2009). In the MFM structure, the ferroelectric layer needs to be as thin as 3–4 nm in thickness to ensure a reasonable tunneling current, but such thickness leads to reduced polarization and TER ratio. Additionally, the traps generated by the tunneling current can reduce the memory window. To address this problem, researchers are investigating a metal-ferroelectric-interlayer-metal (MFIM) structure, which introduces an additional thin tunneling layer to the MFM structure. The MFIM structure improves TER by creating an extra energy barrier by inserting an interlayer (IL) with a high bandgap into the silver MFM structure. By separating the tunneling effect and ferroelectric materials, electron tunneling occurs through thin tunneling, and it can take advantage of tunneling optimization and high speed.

As a synaptic device, it has been investigated for its notable advantages, including high-speed switching, multi-value cell operation, and low power consumption attributed to low current levels and operating voltage (Chen et al., 2018; Majumdar et al., 2019; Ryu et al., 2019; Max et al., 2020; Song et al., 2022). In addition, it can operate with low power compared to other synapse devices because it has good endurance characteristics of 10^5^ or more and uses a TER-based current mechanism. Polarization changes to store information, and various states can be implemented by adjusting the polarization of the domains by changing the programming voltage. Ryu et al. (2019) improved the TER ratio by fabricating an HZO-based FTJ with an interfacial Al_2_O_3_ layer added. The reliability of the Synapse device was measured to secure endurance (>10^7^) and retention (10 years) characteristics. Using the incremental step pulse programming (ISPP) 10 μs pulse method, linear and symmetric characteristics were obtained for long-term potentiation (LTP) and long-term depression (LTD). Luo et al. (2022) fabricated an FTJ with a PZT-based metal-ferroelectric-semiconductor (MFS) structure. It was possible to operate in the multilevel state of the (111)-oriented PZT structure due to the ultra-fine polydomain structure and at a very high speed (10 ns). As characteristics of the synaptic device, ISPP pulses were used (10 ns), 256 conductance states, high dynamic range (~100), linearity close to 1, and symmetric characteristics were secured, as depicted in Figure 4L. In addition, it secured the characteristics of a synaptic device with very high endurance (10^9^) and retention (10^4^ s). Goh et al. (2021) fabricated an FTJ with a TiN/HZO/TaN/W structure. The TaN layer prevented diffusion and prevented leakage current, improving the TER value (~100). The endurance (~10^8^) and retention (10 years) characteristics of the synaptic device were secured, and 30 multilevel states were secured through LTP and LTD measurements.

#### 2.1.5 Array demonstration of two-terminal devices

**Figure 5:**
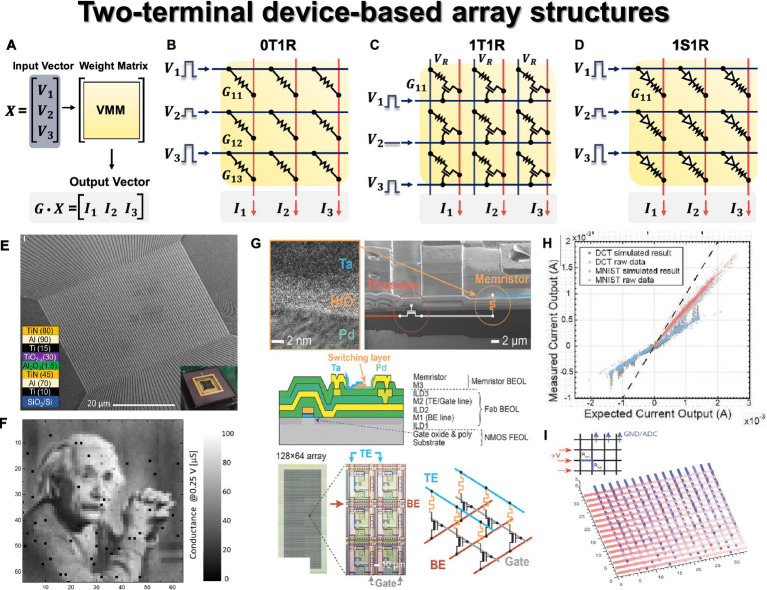
Two terminal device array structures. **(A)** Schematic of VMM operation with input vector, weight matrix, and output vector. **(B)** 0T1R passive crossbar array structure. **(C)** 1T1R array structure. **(D)** 1S1R array structure. Yellow panel indicates the weight matrix implemented by the conductance of two-terminal devices, and red lines indicate the bitline where output currents are obtained through Ohm’s law and Kirchhoff’s current law. **(E)** Scanning electron microscopy image of 64 × 64 0T1R crossbar array. **(F)** Weight tuning results in crossbar array. **(E,F)** Reproduced under the terms of the CC-BY Creative Commons Attribution 4.0 International License (Kim H. et al., 2021), Copyright 2021 Springer Nature. **(G)** Fabricated Ta/HfO_2_/Pd memristor 1T1R array. **(H)** VMM results in 1T1R array. To check the DPE VMM error, circuit simulation (gray) and raw data (colored) from experimentally measured VMM data were compared for both a signal processing application of the discrete cosine transform and neural network inference for the MNIST database. **(I)** Impact of ‘stuck on’ defects and parasitic of 16 × 16 array, resulting in the signal degradation from left to right and from top to bottom. Red lines indicate the rows of 16 × 16 array with applying voltages from left, and blue lines indicate the columns grounded on top, and red dots indicate “stuck on” defects. **(G–I)** Reproduced under the terms of the CC-BY Creative Commons Attribution 4.0 International License (Hu et al., 2018), Copyright 2018 John Wiley & Sons.

The key operation of neural networks is VMM. During inference, large-scale VMM operations need to be efficiently implemented on hardware using a crossbar array (CBA) structure. The CBA structure is inherently suitable for VMM based on synaptic devices (Gao et al., 2016, 2017). As depicted in Figure 5A, input voltages ( $V_{j})$ encoded from input data are applied to each row (*j*th) or each WL of the CBA. Through Ohm’s law at each device cell ( $I_{ij}=V_{j}\times G_{ij}$ ) and Kirchhoff’s current law ( $I=\sum I_{ij}$ ), the BL currents are obtained as a result of VMM operation. These currents contribute to the weighted sum within the activation neuron circuits. Furthermore, to account for negative weights in the CBA, a commonly employed technique is the use of a differential pair, involving a pair of devices for each weight.

The most basic two-terminal device-based CBA structure is the 0T1R passive array, consisting of memristor elements (1R) in each unit cell. As depicted in Figure 5B, the passive CBA structure positions a two-terminal element at the crosspoint where WL and BL intersect perpendicularly. In this configuration, where two-terminal elements are located in parallel, an appropriate bias scheme is necessary to program and read only the selected cell. Accurate array operation for the selected cell is crucial for efficient performance of the entire HW-ANN, including inference accuracy, speed, and power efficiency. The half-V scheme represents a standard biasing method commonly employed in passive CBA. It aims to enhance cell selectivity by applying full bias (*V*) to the WL of the selected cell and 0 V to its BL. Meanwhile, unselected WLs and BLs receive half of the full bias (*V*/2). Specifically, when reading a certain cell, *V*_read_ is applied to the top electrode (WL, row), while 0 V is applied to the bottom electrode (BL, column). However, for the remaining unselected cells, half-*V*_read_ (*V*_read_/2) is applied to the remaining rows and columns, ensuring that the voltage across those cells remains at 0 V. For instance, when reading the first column in Figure 5B, *V*_read_ is applied to all rows, 0 V is applied to the first column, and *V*_read_/2 is applied to the rest of the columns simultaneously. In this scenario, other unselected cells located in the same row or column as the selected cell experience *V*_read_/2. The better the device *I*-*V* non-linearity (or *I*-*V* selectivity) that ensures low OFF current for unselected cells compared to the ON current for selected cells, the more effectively the off-switch operation can be carried out on those cells. This approach enables parallel operations with the weight matrix implemented in the CBA when a specific input voltage vector is applied. However, if sufficient *I*-*V* selectivity (*I*@*V*_read_/*I*@*V*_read_/2) is not guaranteed, passive arrays with high integration and low process costs encounter issues such as unwanted current flow in unselected cells (sneak current issue) and read errors during the half-*V*_read_ scheme (half-select disturbance). This results in challenges in high-precision weight programming/reading in passive arrays. Nonetheless, this can be mitigated with an additional non-linear selection device (referred to as an extra access device, selection device, or series device), including transistor, diode (with unipolar memristor), or selector (with bipolar memristor) devices.

A 1T1R structure is one of the active arrays having a transistor with a gate terminal that provides switch and selective operation for the selected device, as shown in Figure 5C. Typically, a two-terminal memristor is deposited on the drain end of a transistor co-integrated through a mature CMOS integration process. Due to the maturity and controllability of the CMOS process and transistor operation, many neural network applications have adopted the 1T1R-based CBA structure (Yu et al., 2016; Yao et al., 2017; Hu et al., 2018; Cai et al., 2019; Xue et al., 2020; Yao et al., 2020). Excellent weight programming is possible for the desired cell, but the 1T1R structure has a tradeoff between the scaling difficulty of the transistor (at least 6*F*^2^ when W/L = 1) compared to the passive 0T1R structure with 4*F*^2^ and difficulty in 3-D stackability. To resolve this tradeoff, the need for a two-terminal synaptic device and a serially stackable two-terminal selection device emerged. A 1D1R structure addresses this need, where a diode, a representative non-linear device, is utilized as a selection device. The diode stack can be deposited in series with the memristor, each stackable on top, solving the low integration density of 1T1R (Kim et al., 2013; Gül, 2019; Li et al., 2021). Additionally, the rectifying characteristic of diode can guarantee *I*-*V* selectivity, but resistive devices connected in series must have unipolar characteristics, enabling set/reset switching for voltage of one polarity. However, the unipolar memristor device faces challenges such as low program margin (weight precision) due to a narrow switching voltage window (Huang et al., 2011). Additionally, in the case of 1D1R, reverse bias is applied to unselected WL/BL, which imposes stricter requirements on non-linearity factors compared to 1S1R.

A 1S1R configuration, depicted in Figure 5D, involves stacking selector materials and a memristor in series, effectively alleviating the sneak current problem. This configuration combines the advantages of both active and passive arrays, meaning that it possesses the benefits of read/write selectivity while improving integration density. The selector is expected to perform a role similar to that of a transistor in a 1T1R configuration, exhibiting high non-linearity in its *I*-*V* characteristics and offering electroforming-free behavior and scalability. The selector exhibits bipolar switching characteristics, with various material stacks studied to fulfill this function (Wong et al., 2012; Burr et al., 2014; Aluguri and Tseng, 2016; Li et al., 2021; Woo et al., 2022). From a stack perspective, these are referred to as two-terminal selector ‘materials’, and from a device perspective, they can be expressed as a ‘bidirectional diode’ (Jeon et al., 2024). To distinguish it from the diode used for the unipolar memristor device in the 1D1R, selector material compositions with bidirectional diode characteristics are included in the 1S1R configuration. Representative types include ovonic threshold switching (OTS) with high compatibility of PCM devices (Kau et al., 2009; Lee et al., 2012, 2013; Song et al., 2015; Chen et al., 2016; Velea et al., 2017; Hua et al., 2019), mixed ionic-electronic conductors (Gopalakrishnan et al., 2010; Burr et al., 2012; Shenoy et al., 2014; Luo et al., 2015), metal–insulator-transition (Lee et al., 2007; Son et al., 2011; Kim S. et al., 2012; Lee et al., 2015; Cha et al., 2016), tunnel barrier (Lee et al., 2012; Choi et al., 2016; Upadhyay et al., 2020), and field-assisted super-linear threshold switching materials (Jo et al., 2014, 2015).

The 1S1R structure requires a balance between the conductivity of the selection device and the memristor device. Devices with excessively high conductivity may struggle to function effectively as selectors, while those with low conductivity may require higher operating voltages and could reduce the programming window, complicating the design process for optimized read/write voltage margins (Wong et al., 2012; Li et al., 2021). Furthermore, additional selector devices or materials may pose challenges in terms of material optimization and fabrication compatibility with the memristor device. In response to these challenges, there is active research into self-rectifying memristor (or self-rectifying cell) passive arrays. These arrays eliminate the need for extra access devices for memory elements found in existing 0T1R passive arrays. The memristor cell itself exhibits *I*-*V* non-linearity characteristics and offers an alternative to the area overhead of 1T1R structures and the design complexity of 1S1R configurations (Kim K.-H. et al., 2012; Hsu et al., 2013; Kim et al., 2016; Li and Xia, 2019; Sun et al., 2019; Jeon et al., 2024). However, there is a need for further research into CMOS-compatible selector-based large-scale 1S1R crossbar array structures.

The fabrication of a large-scale hardware synaptic device-based array, a 64 × 64 Al_2_O_3_/TiO_x_ RRAM passive array, is presented (see Figure 5E) for *ex-situ* training (Kim H. et al., 2021). By utilizing low-temperature processes, high yield of 99% is achieved, ensuring the array’s high potential for synaptic operations. Additionally, three voltage-programming techniques (bound reducing, high-voltage devices aware, and shifting conductance) are proposed for precise state writing within the array, and the result of weight import (write) on CBA is shown in Figure 5F. In particular, the passive array structure has been shown to address the issue of half-select disturbance that arises in passive array configurations. This research demonstrates the potential for mitigating half-select disturbances in passive array structures and validates the robust operation of the passive array. These techniques take into consideration hardware characteristics, securing the required multilevel state in synaptic devices by ensuring a reliable weight import, a critical operation in *ex-situ* training. Hu et al. (2018) fabricated a 128 × 64 Ta/HfO_2_-based 1T1R array (Figure 5G) and experimentally demonstrated its impressive performance in analog computing for the VMM operation in a CBA. In Figure 5H, the linear relationship between VMM experimental raw data and ideal circuit simulation, including circuit parasitic, was shown for both discrete cosine transform signal processing applications and neural network inference for the MNIST dataset. It suggests a close match between the simulation-based VMM results and the measured one from the fabricated array-based VMM, demonstrating 6-bit precision, re-programmability, and stable operation. In addition, it includes simulations displaying the influence of the impact of faulty cells on VMM performance, as shown in Figure 5I. This article showcases the potential of a memristor CBA-based Dot Product Engine (DPE) for analog computing. Li et al. (2022) demonstrated the potential of HW-ANNs using a 2-D material, HfSe_2_-based CBA. Traditional bulk-based and transition metal oxide (TMO)-based memristor devices suffer from limited resistive switching ratios and challenges related to variations. To address the hard-breakdown issue caused by the forming voltage in bulk material-based RRAM devices in CBAs, a 3 × 3 memristor array was fabricated using polycrystalline HfSe_2_ thin films with defects and dislocations. Through stable control of conductive filaments via defect paths, the HfSe_2_-based 3 × 3 array was experimentally shown to enable hardware neural network operations in *ex-situ* training for edge extraction.

Giannopoulos et al. (2018) experimentally demonstrate the VMM performance on the projected PCM array as 8-bit precision and low power (60 nW). A single-layer neural network implemented on a 10 × 3 GeTe-based projected PCM array successfully performed inference without errors even under varying external temperatures. The projected PCM devices exhibit much weaker field dependency compared to conventional PCM (Koelmans et al., 2015), thereby resolving the conductance drift issue caused by structural relaxation in the amorphous state of conventional PCM. Consequently, they enable more accurate VMM. Figure 4L shows the VMM results obtained from 2,000 experimental runs of 4 × 3 VMM, which closely approximates the performance of 8-bit fixed-point arithmetic with the inclusion of temperature compensation techniques. A three-layer neural network consisting of 164,885 synapses was experimentally implemented using a 500 × 661 1T1R PCM array for *in-situ* training (Burr et al., 2015). Each synapse utilized two PCM devices (*G*^+^, *G*^−^) as a differential pair, and the weighted sum operation was performed by the software-based neurons with sense amplifiers to process the column currents. The measurements obtained from the hardware implementation were compared with simulation results based on parameter values, and a close match was observed, confirming the predictability of the array’s behavior and consideration of non-ideal effects.

An experimental implementation of *ex-situ* training was achieved using a 64 × 64 1T1MTJ CBA-based hardware neural network (Jung et al., 2022). To address the power consumption issue in large-scale arrays caused by the low resistance of MRAM, a new cell-based CBA was fabricated, proposing an energy-efficient synaptic hardware system. The 1-bit cell, composed of two complementary devices, represents the 1-bit state with the combination of FET’s gate voltage and the state of MTJ. For conventional VMM operations, Kirchhoff’s current law was used to calculate the current sum. However, by measuring the RC time delay, the new structure determines the neuron output based on the resistance sum of each column. The time taken to charge the capacitor at the end of each column to *V*_ref_ varies depending on the resistance of each column, allowing VMM operations to be performed based on the time difference based on the CBA. Berdan et al. (2020) fabricated an FTJ in which thin SiO_2_ was added to the ferroelectric layer doped with SiO_2_ to HfO_x_ and confirmed the VMM operation in a neuromorphic system in a passive 5 × 5 crossbar array. An FTJ array without a selector was fabricated, and analog voltage-amplitude VMM operation was demonstrated using the non-linear and very low current characteristics of the FTJ. The non-linear *I*-*V* characteristics were linearized using a word line driver, and the very linear *I*-*V* for input voltage in 16 conductance states was confirmed through experiments. MM operation was performed for 100 inputs by adjusting the state with different weight maps, and a very accurate result was obtained with an error of 0.77% between the value calculated through effective conductance and the actual VMM operation.

### 2.2 Three-terminal devices

#### 2.2.1 Flash memory

**Figure 6:**
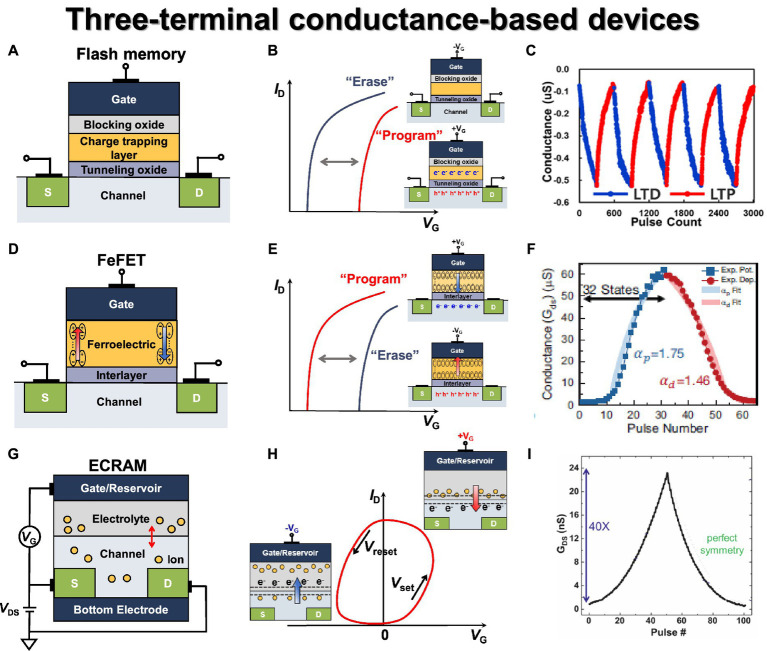
Three terminal conductance-based synaptic devices. **(A)** Flash memory basic structure. **(B)** Flash memory transfer curve. **(C)** Conductance modulation on flash memory under incremental step pulse programming (ISPP). Reproduced with permission (Zhou et al., 2022), Copyright 2022 IEEE. **(D)** FeFET basic structure. **(E)** FeFET transfer curve. **(F)** Conductance modulation under ISPP. Reproduced with permission (Jerry et al., 2017), Copyright 2017 IEEE. **(G)** ECRAM basic structure. **(H)** ECRAM transfer curve. **(I)** Conductance modulation on ECRAM under identical pulses. Reproduced with permission (Tang et al., 2018), Copyright 2018 IEEE.

Flash memory is one of the most representative and successfully commercialized three-terminal non-volatile memory devices. It has gained popularity due to its scalability, high reliability (Mizoguchi et al., 2017), and multilevel capabilities (Wang F. et al., 2020). However, both charge-trapping layer (CTL)-based and floating gate (FG)-based devices suffer from the drawback of requiring high voltage during program and erase operations, which can lead to a decline in endurance characteristics (Lee et al., 2003). As technology scales down, researchers have investigated solutions like 3-D NAND flash or closer cell spacing to ensure better scalability and to overcome cell-to-cell interference (Jang et al., 2009; Compagnoni and Spinelli, 2019). To achieve better scaling and performance, the flash memory structure has evolved from the traditional MOSFET to a configuration involving blocking oxide/charge trapping layer/tunneling oxide, as illustrated in Figure 6A. In the past, FG was used as the charge-trapping layer, but this approach became impractical due to cell-to-cell interference as scaling continued (Lu, 2012). Thus, insulator materials with high trap density like Si_3_N_4_ and HfO_2_ have been adopted as CTLs (Jung et al., 2006; You and Cho, 2010). Each material offers distinct advantages and disadvantages, with HfO_2_ having good memory window but poor retention, while Si_3_N_4_ has excellent retention due to the absence of shallow traps.

In general, flash memory device can be integrated into NAND and NOR array structures based on the connection method, leading to a distinct program/erase method for each cell (Bez et al., 2003). NOR flash enables parallel computation for ANN applications, but it suffers from low area efficiency. On the other hand, NAND flash exhibits excellent area efficiency but lacks parallel summation capability due to its serial connection structure. As a result, to read the entire current of one bit line, the string cell must be read sequentially, leading to a significant disadvantage in terms of speed. Both NAND and NOR structures utilize Fowler–Northeim (F-N) tunneling for the erase method. However, they differ in their programming methods. In NOR flash, a high positive voltage applied to the gate and drain *via* channel hot electron injection causes electrons to move to the CTL in the drain pinch-off region. In NAND flash, a strong positive voltage applied to the gate through F-N tunneling bends the tunneling oxide, enabling electron injection into the CTL. When electrons are accumulated in the charge-trapping layer, the formation of the inversion layer is hindered. As a result, the threshold voltage is increased, and the threshold voltage is decreased in the opposite case, as shown in Figure 6B.

As a synaptic device, it offers significant advantages for analog computing, such as excellent endurance and retention characteristics, a wide memory window enabling numerous states, large on/off ratio, and good linearity. Zhou et al. (2022) demonstrated the conductance modulation of 3-D NAND differential pair with identical LTP and LTD pulses, as shown in Figure 6C. Due to the block erase operation in the NAND flash architecture, the synaptic weight was decreased or increased by simply programming one of the devices within the differential pair. However, in the NAND flash structure, cells are connected in series, and multiple cells are connected to a single WL. Due to the structural features of the NAND flash array, performing an inference operation may alter the weights of unintended cells due to the read and pass voltage. Therefore, it is essential to assess the disturbance characteristics caused by read and pass voltages. The repeated weight modulations were performed using an identical pulse (12 V, 13 μs), and excellent disturbance characteristics were confirmed for read (10^8^) and program (10^6^) for reliability characteristics as a synapse device. Lee et al. (2019b) employed a 2-D NAND flash string fabricated in the industry using 26 nm technology and confirmed the characteristics as synaptic devices. By changing the drain voltages, 30 multilevel states were secured, and the LTP and LTD characteristics were repeatedly checked using identical pulses (14 V, 100 μs). The retention (>10^4^ s) and conductance response (after 1 k endurance cycling) were checked in 30 states for reliability characteristics.

#### 2.2.2 Ferroelectric field-effect transistor (FeFET)

FeFET has a three-terminal structure with a ferroelectric layer as the gate dielectric. FeFET controls the polarization direction of the ferroelectric layer through the gate voltage, and the threshold voltage is modulated in the opposite direction compared to flash memory, as depicted in Figure 6D. When a positive voltage is applied, the threshold voltage is lowered due to polarization and positive charge on the channel side, whereas the threshold voltage increases when a negative voltage is applied, as shown in Figure 6E. Unlike a flash memory device, which necessitates high voltage for F-N tunneling during program and erase operations, FeFET takes advantage of polarization induced by low switching voltage, resulting in lower power consumption. While perovskite materials have been studied for their good endurance and high-speed operation, hafnium oxide-based ferroelectric layers have gained attraction due to good CMOS process compatibility and scaling down (Böscke et al., 2011). FeFETs can be fabricated by replacing the gate dielectric layer with HfO_x_ or HfZrO_x_ (HZO) layers through low-temperature atomic layer deposition (ALD), following the conventional CMOS process flow (Ali et al., 2018; Mulaosmanovic et al., 2021).

Unlike the early Si channel-based MFS structure of the FeFET device (Yoon et al., 1999), the annealing process used to create the orthorhombic phase of HZO layer has led to the formation of a SiO_2_ interlayer between the ferroelectric gate dielectric and Si channel. When an intrinsically grown SiO_2_ interlayer is formed, applying voltage to the gate results in most of the electric field being confined within the SiO_2_ layer because of a lower dielectric constant (3.9) compared to HZO (20–40). This disparity in dielectric constants can potentially lead to charge trapping/de-trapping, interface reaction, and inter-diffusion issues (Tokumitsu et al., 2000; Anderson et al., 2018), which can contribute to poor endurance, retention, and a small dynamic range of FeFETs. To address the interface reaction and diffusion issues observed in the MFS structure, the metal-ferroelectric-insulator–semiconductor (MFIS) structures have been explored with the insertion of an interlayer between the ferroelectric layer and the Si layer (Mueller et al., 2013; Mulaosmanovic et al., 2017; Ni et al., 2018). The interlayer offers the advantage of preventing interdiffusion and interface reactions, thus reducing gate leakage current. When utilizing the MFIS structure, the ferroelectric layer and the dielectric interlayer form a configuration where capacitors are connected in series. This structure design leads to a decrease in the potential difference across the ferroelectric layer, posing a challenge that necessitates higher voltage for programming and erasing operations. To optimize the gate voltage applied to the ferroelectric layer, the magnitude of the voltage applied to the interlayer can be decreased by employing a high-k material for the interlayer, leading to extensive exploration into investigating suitable stack configurations. The MFMIS structure enhances the MFIS design by incorporating a floating gate metal layer between the ferroelectric layer and the Si substrate. The top MFM and bottom MIS components are independently designed, which effectively mitigates voltage drop issues in the MFM capacitor. This leads to a reduction in the operating voltages required for program and erase operations, addressing a pre-existing concern and significantly improving data retention characteristics.

FeFET emerges as a strong candidate for a synaptic device given its advantages, including low operating voltage and power consumption. Additionally, it exhibits effective conductance control, a high on/off ratio, a minimal sneak path, and rapid operation speed (Mulaosmanovic et al., 2017; Jerry et al., 2018). Jerry et al. (2017) fabricated an HZO-based MFIS-structured FeFET and demonstrated multilevel characteristics of high dynamic range (45) and 32 conductance state levels by using multi-domain polarization characteristics. The linearity characteristics were verified by using three methods of changing identical, ISPP, and pulse width modulation, and the linear and symmetric weight update characteristics were secured with ISPP pulses (75 ns, 50 mV step), as shown in Figure 6F. Dutta et al. (2020) fabricated a BEOL-compatible FeFET capable of 3-D integration with In_2_O_3_ (IWO) channel and HZO layer. A wide memory window of 1.2 V, a fast write speed of 100 ns, endurance cycle (>10^8^), and memory retention characteristics (>10^3^ s) were confirmed through gate voltage pulses. In addition, uniform cycle-to-cycle variation was secured for the state of 2 bits according to the program voltage. Kim M.-K. et al. (2021) fabricated a FeFET using an HZO ferroelectric layer and an IZTO channel. The conductance modulation characteristics were confirmed through the gate voltage, and the on/off ratio (>10) and endurance characteristics (>10^7^) were secured. To obtain linear and symmetric characteristics, the ISPP method was used to secure 64 conductance states and symmetric characteristics of 0.98 and 1.1795.

#### 2.2.3 Electrochemical random access memory (ECRAM)

Electrochemical RAM (ECRAM) is a non-volatile, three-terminal device operating with electrochemical switching behaviors. It consists of a gate, ionic reservoir, source/drain, electrolyte, and conductive channel, as depicted in Figure 6G (Nitta et al., 2015; Van De Burgt et al., 2017; Jeong et al., 2021; Kang and Woo, 2021; Talin et al., 2022). It is also known as an ion-based synaptic transistor. In ECRAM, the conventional gate dielectrics of three-terminal transistors are replaced with an electrolyte layer. Depending on the applied gate voltage, ions from the electrolyte diffuse into the channel, inducing an electrochemical change that controls the conductance of the channel, as shown in Figure 6H. In general, the electrolyte ions include Li^+^, H^+^, O^2−^, etc., and lithium-ion-based synaptic transistors have been extensively studied (Fuller et al., 2017; Yang et al., 2018). By allowing ions to leave or enter the channel, ECRAM can modulate the conductivity of the channel, enabling it to function as an analog synaptic device. The conductivity of the channel is monitored by applying a fixed voltage to the source-drain voltage during read and write operations. ECRAM exhibits relatively symmetric switching characteristics and low stochasticity due to the control of electrochemical reactions by the amount of charge generated in response to the applied gate voltage.

Tang et al. (2018) demonstrated an ECRAM device based on a WO_3_ channel with LiPON electrolyte and its operational characteristics. By applying positive and negative gate current pulses, the conductance of the WO_3_ channel was modulated through the intercalation of lithium ions, achieving potentiation and depression responses, as shown in Figure 6I. The measured conductance response exhibited excellent symmetry, a large dynamic range (up to 10^3^), and minimal stochasticity, allowing for precise conductance state representation. Furthermore, the ECRAM device demonstrated the endurance characteristics with no degradation, lasting up to 10^5^ pulses. Additionally, the ECRAM device showed high-speed programming capability (down to 5 ns) and scalability potential, indicating its promising prospects as a synaptic device. Kim et al. (2019) demonstrated a metal oxide ECRAM array based on WO_3_ channel, HfO_2_ electrolyte, and metal oxide reservoir stack, and both array operations and stochastic update algorithm-based weight update characteristics were experimentally presented. The fabricated devices exhibited reliable synaptic characteristics, including retention of over 14 h after programming, endurance of 20 million pulses, and an on/off ratio of 2. The metal oxide ECRAM-based 2 × 2 array was configured with the drain terminals of each device connected in one row and the source terminals connected in one column, and the half-*V* scheme was employed for weight updates. The array was trained using a linear regression problem, and the learning algorithm tuned the weights toward the target values. As the training progressed, the weight values converged to target values, and the error with respect to the epoch approached zero, as demonstrated through experimentation.

#### 2.2.4 Array demonstration of three-terminal devices

**Figure 7:**
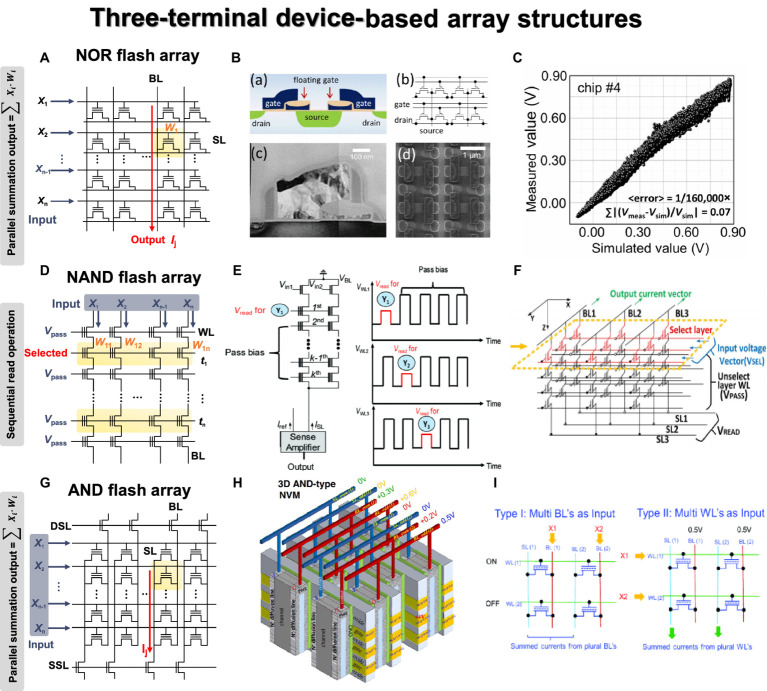
Three terminal device array structures. Yellow panel indicates the weight matrix implemented by conductance of three-terminal devices, and red lines indicate the bitline where output currents are obtained through Ohm’s law and Kirchhoff’s current law. **(A)** NOR flash array. **(B)** Analog VMM circuit schematic using floating gate NOR flash structure. **(C)** Simulated and measured VMM results for 10,000 inputs. **(B,C)** Reproduced with permission (Guo et al., 2017), Copyright 2017 IEEE. **(D)** NAND flash array. **(E)** Circuit diagram and bias scheme of 3-D NAND array architecture for VMM operation with sequential read method. Reproduced with permission (Lee et al., 2019a), Copyright 2019 IEEE. **(F)** The circuit diagram of VMM operations in 3-D NAND array structure. For the selected layer, the selected input voltage vector is applied to WL, and a pass voltage is applied to WL of the remaining layers. *V*_read_ is applied to SL, and GND is applied to BL to read the BL current. Reproduced with permission (Wang P. et al., 2018), Copyright 2018 IEEE. **(G)** AND flash array. **(H)** Schematic diagram of 3-D AND-type NVM. **(I)** Two VMM methods proposed in the 3D AND flash array structure. Method 1: Insert analog input into BL to read current from SL. Method 2: Apply binary input to WL to enable high-density, fully connected operation. **(H,I)** Reproduced with permission (Lue et al., 2018), Copyright 2018 IEEE.

Most three-terminal devices are integrated into flash memory arrays, and representative structures are NOR, NAND, and AND flash arrays. Additionally, some studies have demonstrated the use of 3-D stacked arrays, considering the commercialized 3-D NAND flash memory. Figure 7A illustrates the structure of a 2-D NOR flash array and the VMM operation mechanism. Similar to the memristor CBA, each cell is connected in parallel, enabling simultaneous VMM operations. When input signals are applied to WLs, drain current with constant bias can be summed along with the BL or source line (SL) direction. The input signals can also be applied to BLs, and current summations occur along with SLs. Guo et al. (2017) employed embedded NOR flash memory technology for neuromorphic classifiers, as shown in Figure 7B. For analog computing, the synaptic devices in the array exhibited near analog-grade weight levels achieved by the weight-tuning process, including a verification step. The accurate VMM results were experimentally demonstrated with 10,000 CIFAR-10 test images, as depicted in Figure 7C. The reliable classification operation was also verified by comparing the relative changes of the output voltages for all 10,000 MNIST test images between the originally measured one and those measured 7 months later.

In contrast to NOR flash array, NAND flash structure connects cells in series to form a string, as shown in Figure 7D. This series connection prevents parallel current summation, allowing only one cell to be read at a time. To read the state of one cell, pass voltage must be applied to all unselected cells in the same string to function as wires. During program or erase operations, a high voltage is applied to the same page due to the shared WL structure, and an inhibit voltage is necessary to prevent disturbance. Because of this structure, simultaneous VMM operation is challenging in NAND flash array. The input signals can be applied to either WL or BL, while the other one is biased. In both scenarios, however, each cell needs to be read sequentially due to the serial connection of NAND flash string. Lee et al. (2019a) presented a method for implementing the XNOR operation using a 2-D NAND string within the group. Figure 7E shows the process of performing VMM operation on a 2-D NAND string, along with the distribution of string current output based on the XNOR operation results. When two cells are utilized as a pair, +1 is an output when the left cell is in the on state. The input signals were applied to the input transistors of the string cells, and the VMM operation was executed while sequentially reading the WLs. The distribution of outputs +1 and − 1 confirmed the proper execution of the XNOR operation based on the state of each cell.

This issue can be addressed with a 3-D NAND flash structure, where each string is connected in parallel through BL. This structural feature allows the string current to be summed along the BL direction, enabling simultaneous VMM operations (Wang P. et al., 2020). The VMM method slightly varies depending on the planes within the 3-D-based structure used for weight mapping. In addition, the technologically matured 3-D NAND flash structure offers significant advantages for implementing large-scale DNNs with numerous synapse weights. Wang P. et al. (2018) demonstrated a method for VMM operation based on 3-D NAND flash architecture, as shown in Figure 7F. The weight matrix was mapped on one WL layer (XY plane), so the selection voltage was needed for the selected WL, while pass voltage was applied to the unselected WLs. The input signals were applied to the SL in the form of a read voltage so that string current could be summed along the BL. The result of VMM calculation performed in the 256 × 256 × 8 structure was demonstrated with SPICE simulation, showing a linear BL current increase as the number of cells increased. Kim I.-J. et al. (2023) developed a 3-D NAND structure using FeFETs and conducted VMM operations with 4 × 2 images. The fabricated 3-D Fe-NAND structure demonstrated various electrical characteristics, and array operations were successfully verified. Like traditional NAND flash, the pass voltage of 2 V was applied to unread cells, while −5 V pulse was used for erasing, and a program pulse of 4 V was applied to the selected cell with 0 V. A voltage of 2.5 V was applied to the BL for the unwanted string. Through repetitive device measurements, program, erase, and inhibit characteristics were confirmed to work effectively. The device also exhibited reliable retention characteristics (10^6^ s) and endurance operation (10^6^) in each state, proving its usability as a synapse device. As a synapse array, the input signal was applied to BL in the 3-D NAND structure, and string current was summed to the SL line as a result of the multiplication between the BL voltage and the cell conductance.

AND flash array is also one of the potential candidates as a synapse array for three-terminal devices, as shown in Figure 7G (Jang et al., 2020; Lue et al., 2020; Seo et al., 2021). Its VMM operation is very similar to that of the NOR flash array. The input signals can be applied to either WL or BL, and each cell current can be summed along with SL. Since each cell is integrated in parallel along with the BL and SL directions, parallel current summation and VMM operations can be conducted. Due to the parallel BLs and SLs, sneak paths can be prevented during programming, and 3-D array integration is in a structure similar to that of 3-D NAND flash architecture with common drain and source plugs. Lue et al. (2018) presented 3-D AND-type array for in-memory VMM computations, as shown in Figure 7H. The current summations were conducted along with the SL as a result of the multiplication between the BL voltage and the device conductance. In addition, two VMM methods were proposed for the 3-D AND flash array depending on whether WL or BL is used as input signal, as shown in Figure 7I. The former method enabled high-resolution operation because analog input was inserted into BL to read current from SL. The latter method enabled high-density and fully connected operation by applying binary input to WL.

Chen et al. (2023) recently fabricated a 10 × 10 YSZ/WO_x_-based ECRAM array and successfully implemented a mushroom classification task in 10 × 5 × 2 bilayer neural network through *in-situ* training. This experiment represents the largest-scale implementation of an ECRAM array to date, surpassing previous demonstrations limited to single-cell characteristics. Notably, the experiment showcased the feasibility of open-loop programming, leveraging the excellent uniformity, linearity, and symmetry of the fabricated ECRAM devices. By demonstrating accurate programming performance using a single-voltage PWM pulse, the study highlighted the advantages of open-loop programming, such as reduced complexity and enhanced system efficiency compared to closed-loop schemes (write-verify). This achievement underscores the exceptional characteristics of the ECRAM array and underscores both its potential and the challenges associated with implementing large-scale networks in the future. While recent studies have explored programming schemes to overcome the limited operational characteristics of the device, such methods often entail trade-offs in terms of repetitiveness, time consumption, and energy consumption (Maheshwari et al., 2021). Moreover, these approaches may not be readily applied to other synaptic devices due to the unique characteristics of ECRAM.

### 2.3 Capacitor-based devices

When capacitors are connected in parallel, the total capacitance appears as the sum of individual capacitance (*C* = *C*_1_ + *C*_2_). The total charge induced in each capacitor can be expressed as the sum of the charges of parallel-connected capacitors. Consequently, while the conventional conductance-based neural network produces the current as the outcome of VMM operation, the capacitor-based neural network generates the charge in relation to *C* and *V* as a result of VMM operation (Pershin and Di Ventra, 2014; Zheng et al., 2019; Kwon and Chung, 2020; Luo et al., 2021). Additionally, capacitive devices can be integrated into a 4*F*^2^ CBA due to their two-terminal structure. However, capacitive CBAs exhibit distinct characteristics compared to memristive CBAs. Firstly, IR drop is negligible since the cell resistance is significantly greater than the wire resistance, unlike memristive CBAs, where a serious IR drop can occur in a large-sized array. Due to the open-circuit nature of a capacitor, sneak path current can also be effectively suppressed. Additionally, when using pulse-related inputs, a capacitor-based array can consume less power compared to conductance-based arrays since it only uses dynamic power.

**Figure 8:**
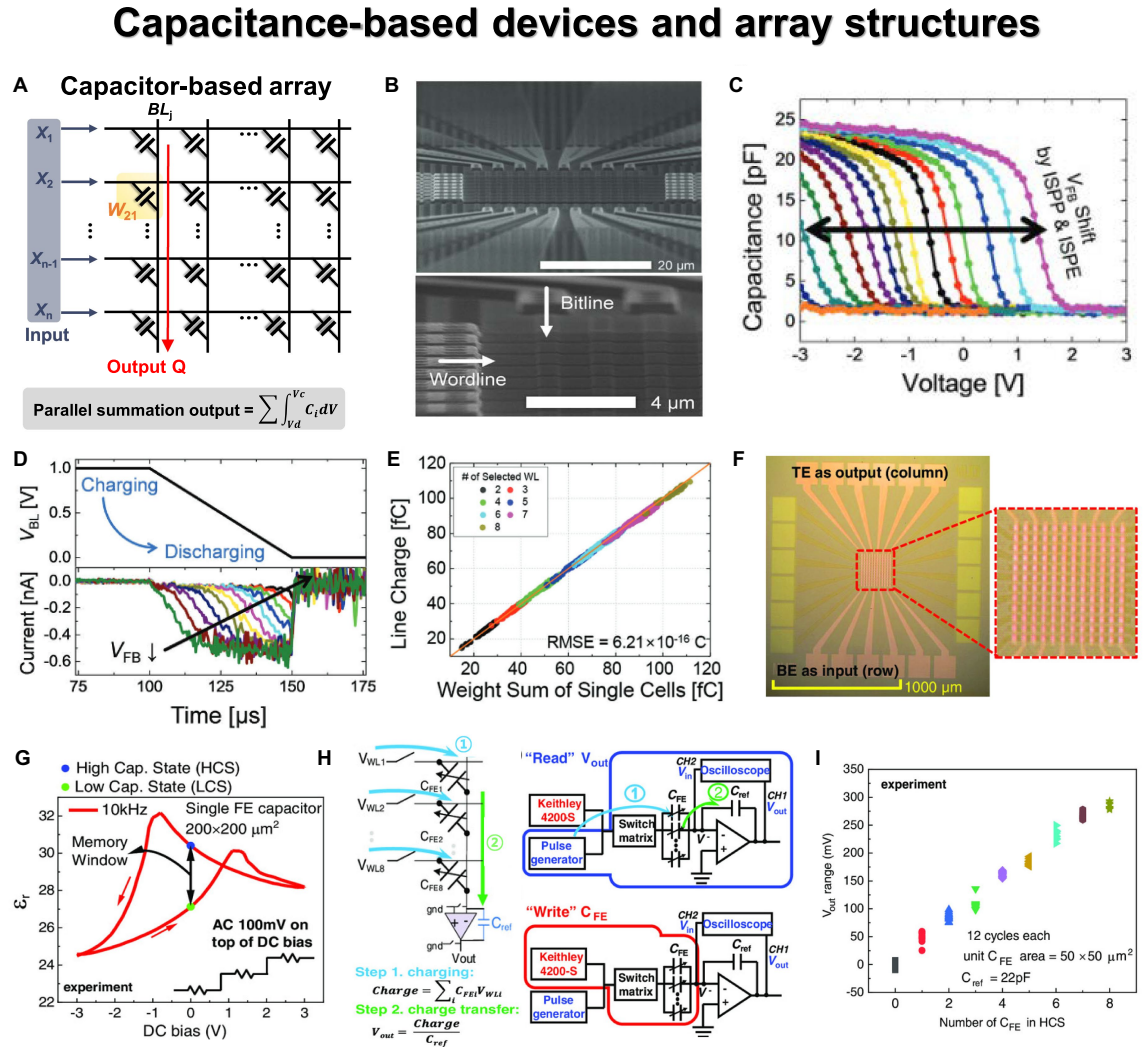
Capacitor-based devices and array structures. **(A)** Basic structure of capacitive crossbar array. **(B)** SEM image of 8 × 16 capacitive crossbar array based on charge trap flash structure. **(C)** C-V characteristics according to program and erase voltage. **(D)** Discharging current characteristics according to the capacitor state. **(E)** VMM operation results measured through the fabricated 8 × 16 capacitive crossbar array. **(B–E)** Reproduced with permission (Hwang et al., 2023). Copyright 2023 WILEY-VCH. **(F)** Optical image of fabricated ferroelectric capacitive crossbar array. **(G)** Physical illustration of the asymmetric *C*–*V* characteristics. **(H)** The weighted sum operation in capacitive crossbar array. **(I)** Voltage output through a weighted sum according to the number of high-capacitance states. **(F–I)** Reproduced under the terms of the CC-BY Creative Commons Attribution 4.0 International License (Hur et al., 2022), Copyright 2022 John Wiley & Sons.

The basic operating principle of capacitive neutral networks is illustrated in Figure 8A, which shows the schematic of the capacitive crossbar array. In general, the operation method proceeds in two stages. The first step is to charge the capacitive devices. When a voltage pulse is applied to the WL, the accumulated charges vary depending on the device state. Then, the stored charges in the capacitor flow through the BL when the discharging voltage is applied. The charges in each cell are added through *Q* = ∑*CV*, where *Q* can correspond to the VMM results. In general, the output voltage is obtained by transforming *Q* through an integrator connected to the BL.

One potential candidate for a capacitor with memory function, known as a memcapacitor, is a metal oxide semiconductor (MOS) capacitor based on a charge trap flash (CTF) cell structure. Since its threshold voltage can be modulated by the trapping and de-trapping of electrons, the capacitance is adjusted accordingly. Hwang et al. (2023) fabricated the 8 × 16 MOS capacitor arrays with Ti/Al_2_O_3_/Si_3_N_4_/SiO_2_/Si (TANOS) charge trap flash structure as shown in Figure 8B and experimentally demonstrated VMM operations. Employing program and erase schemes based on the F-N tunneling mechanism like conventional flash memory, multilevel characteristics of 4-bit were confirmed, as shown in Figure 8C. Transitioning the applied voltage from the charging voltage (*V*_c_) to the discharging voltage (*V*_d_) resulted in varying levels of discharging current and accumulated charge, dependent on the threshold voltage of the MOS capacitor, as depicted in Figure 8D. The operating principle of the capacitor crossbar array is as follows: When the charging transistor is activated, *V*_c_ is applied to the BLs, and the transistor of the selected WLs initiates charging. Unselected WLs remain in a floating state, with the cells remaining uncharged. Subsequently, when the charging transistor is turned off and the discharging transistor is turned on, the BL voltage shifts from *V*_c_ to *V*_d_, inducing the corresponding charge and discharging current through the BL. The VMM operations were also validated using a randomly generated weight distribution, achieved by adjusting the program and erasing voltages. The excellent correlation value confirms the accurate execution of the VMM results, as shown in Figure 8E.

Ferroelectric capacitor is also another candidate for capacitive neural network, and a lower switching voltage can be employed compared to the MOS capacitor based on CTF cell. Luo et al. (2020) fabricated a non-volatile and tunable MFM capacitive device based on a TiN/HZO/TiN stack. It was explained that the non-volatile capacitance arose from oxygen vacancy accumulation at the bottom electrode. The oxygen vacancies resulted in asymmetric number of domain walls (DWs) for positive and negative biases, subsequently inducing distinct capacitance states within varying polarization conditions. Hur et al. (2022) fabricated a HZO-based ferroelectric capacitive CBA based on the same TiN/HZO/TiN stack, as shown in Figure 8F, and examined its operational characteristics for in-memory computing. The on/off ratio > 110% was achieved by utilizing the DW pinning effect caused by oxygen vacancies at the bottom electrode, resulting in high and low capacitance due to polarization characteristics at DC bias of 0 V, as shown in Figure 8G. As a capacitive synapse device, the device exhibited reliable characteristics, including endurance (10^3^) and retention characteristics (10 years), in both high and low capacitance states. The weighted sum operations were also experimentally demonstrated in a fabricated 12 × 12 array. These operations involved two steps, including charging and charge transfer steps, as illustrated in Figure 8H. Initially, charge was accumulated in the ferroelectric capacitors when a voltage was applied to the WL. Then, the stored charges were transferred to the integrator, and the output voltage was generated accordingly. The VMM operation was verified according to the number of capacitive devices, as shown in Figure 8I.

To address the low on/off ratio observed in the MFM structure, research has explored capacitive synapses using the MFS stack. In the conventional MFM structure, the capacitance values in both states are very small, making it susceptible to noise and variations (Yu et al., 2023). Kim T.-H. et al. (2023) validated the operation of capacitive synapses using the MFS-structured FeFET. They varied the program voltage while keeping the erase voltage fixed to confirm multilevel operation and the on/off ratio at the read voltage. Applying a positive voltage causes the ferroelectric dipole to orient downward, resulting in a larger capacitance value at the read voltage. Through TCAD simulations, they investigated capacitance values in two different states by varying parameters such as gate area and overlapped area.

## 3 Compensation methods against hardware non-idealities

To implement a hardware-based neuromorphic system comparable in performance to software-based neural networks, considerations at each level—device, array, and device/array interface, overhead in peri-circuits—necessarily must be followed by achieving robust overall system performance. Among them, synaptic devices and arrays form the foundational elements at the bottom level of the neuromorphic system. These fundamental components can give rise to potential performance degradation within the overall system if not addressed adequately. This section focuses on the hardware non-idealities in artificial synaptic devices and arrays, which handle the storage and processing of weight values, crucial information in neural networks. In addition, the compensation methods against the hardware non-idealities are discussed.

**Figure 9:**
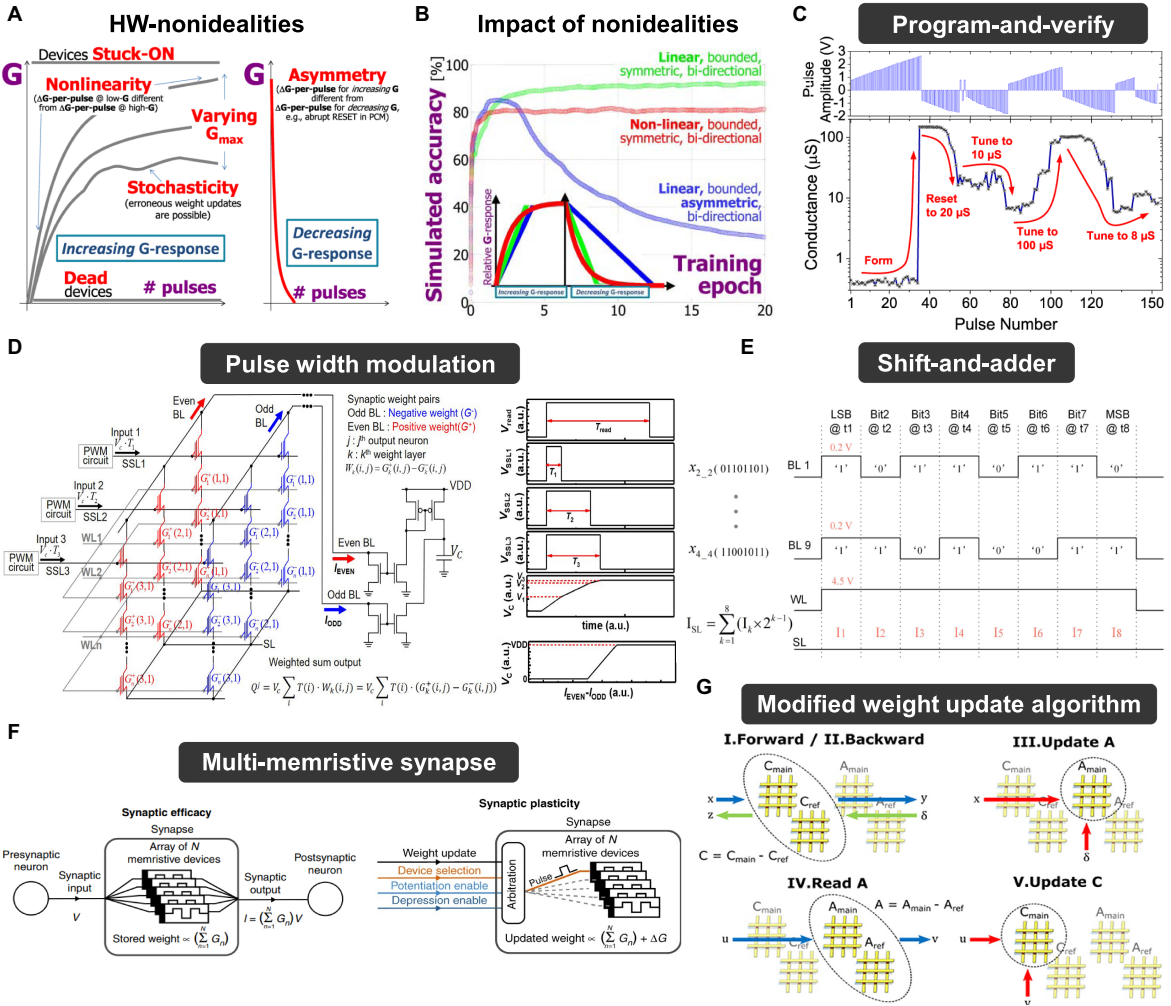
Signal and hardware engineering against non-idealities of synaptic device. **(A)** Various non-idealities of synaptic devices. **(B)** Impact of non-idealities on training accuracy. **(A,B)** Reproduced with permission (Burr et al., 2015), Copyright 2015 IEEE. **(C)** Write-and-verify programming method during weight tuning phase. Reproduced under the terms of the CC-BY Creative Commons Attribution 4.0 International License (Kim M.-K. et al., 2021), Copyright 2021 Springer Nature. **(D)** PWM with the same amplitude and different pulse width depending on the intensity of signal. Reproduced under the terms of the CC-BY Creative Commons Attribution 4.0 International License (Lee and Lee, 2020), Copyright 2020 Frontiers Media S.A. **(E)** Binary pulse scheme encoding 255 levels using shift and adder. Reproduced with permission (Yao et al., 2020), Copyright 2020 Springer Nature. **(F)** Concept of multi-memristive synapse. Reproduced under the terms of the CC-BY Creative Commons Attribution 4.0 International License (Boybat et al., 2018), Copyright 2018 Springer Nature. **(G)** Tiki-taka algorithm with core and auxiliary system composed of main array and reference array. Reproduced under the terms of the CC-BY Creative Commons Attribution 4.0 International License (Onen et al., 2022), Copyright 2022 Frontiers Media S.A.

Imperfections within synaptic devices and array structures can lead to lower neural network learning performance, as shown in Figures 9A,B (Burr et al., 2015; Zhang W. et al., 2019; Joksas et al., 2020; Lim et al., 2021). The non-idealities can arise from both synaptic devices themselves (non-linearity, asymmetry, variation, noise, etc.) and array structures (IR drop, read/write disturbance, sneak path current, interference, etc.), necessitating methods to compensate for or mitigate their impacts. Once the synaptic devices and arrays are fabricated, it is significantly difficult to change or suppress the intrinsic non-ideal factors they possess. As the network size increases, the size and analog characteristics of the required synaptic arrays become more stringent. However, even with unavoidable non-idealities, a flexible approach for compensation is needed. To address the performance degradation of hardware-based neural networks caused by non-ideal characteristics within the devices, various compensation approaches have been proposed. In this section, we explore various approaches designed to alleviate the impact of hardware non-idealities on network performance. These approaches fall into two main categories: signal and hardware engineering, which entail methods implemented at the hardware level; and SW-HW co-optimization, wherein ideal neural networks are benchmarked against software baselines.

### 3.1 Hardware and signal engineering

#### 3.1.1 Program-and-verify methods

During the weight import stage, which is the primary phase of *ex-situ* learning, achieving precise weight writing might be challenging due to conductance variations or malfunctioning devices, leading to a potential degradation in inference accuracy. To achieve accurate weight transfer, a program–verify algorithm (also known as the feedback programming method or closed-loop programming method) is utilized, and, as can be seen in Figure 9C, the set pulse and reset pulses are adjusted while checking the conductance states to reach the target conductances. This tuning algorithm is designed to approach the target conductance as closely as possible by employing read pulses between switching voltage pulses (Alibart et al., 2012; Kim H. et al., 2021). If the conductance state is lower than the target, a set pulse is applied to increase the device conductance; conversely, a reset pulse is used to decrease the device conductance if the conductance state is higher than the target. This iterative process ensures that the conductance falls within the acceptable range, where inter-state overlap is avoided, thus enabling precise state programming. Incorporating a tunable parameter known as the tuning margin provides finer control, and increasing the number of applied pulses compensates for tuning inaccuracies arising from variations in the degree of non-idealities.

Kim T.-H. et al. (2022) successfully transferred 3-bit states in TiO_x_-based RRAM using a program–verify algorithm, ensuring no state overlapping. They applied set/reset pulses within a program error margin of 5 μA to observe the process of reaching the target conductance range. Bayat et al. (2018) further improved tuning accuracy by utilizing the feedback programming method, taking advantage of the device’s inherent variation characteristics. This approach involved continuously verifying the states during programming, enabling control of switching threshold variation on a per-device basis. Additionally, they reduced the impact of tuning inaccuracy on performance and increased operational speed by fixing one of the two RRAM devices that constitute a weight to its minimal conductance during programming, thereby minimizing the influence on the actual weight calculation.

Yao et al. (2017) developed a one-layer perceptron neural network with a 320 × 3 configuration using a 1T1R structure, which was employed for face image classification. They investigated the impact of two methods: the program–verify method, which applies pulses until the target conductance is achieved, and the method utilizing a single set/reset voltage pulse. Analyzing the effects of these methods on 255-level normalized conductance, they observed that, after training, the weights tended to concentrate at lower conductance levels when the write-verify method was employed. When considering input images with added noise components, the write-verify method exhibited better learning accuracy (88.08%) compared to not using it (85.04%), on average. The write-verify approach also demonstrated faster convergence, reduced energy consumption, and higher accuracy due to the fewer required iterations.

#### 3.1.2 Input encoding schemes

To enable effective analog computing, it is crucial to establish a linear correspondence between current and voltage, especially during VMM operations. In general, two approaches are employed to interpret input as an analog signal. One method involves adjusting the voltage magnitude while maintaining a constant pulse width, whereas the other entails modifying the pulse width while keeping the voltage magnitude constant. In the case of altering the voltage magnitude, the linear relationship between applied voltage and devices is mandatory to ensure precise VMM operation. However, most of the non-volatile memory devices under investigation find it challenging to exhibit the necessary linear relationship, which can result in inaccuracies in the output signals. Achieving this linearity can be challenging due to the non-linear *I*-*V* characteristics inherent in non-volatile memory devices.

Lee and Lee (2020) utilized the pulse width modulation with a fixed amplitude, where the pulse width was modulated to express analog-grade input signals, in 3-D NAND flash array as shown in Figure 9D. By employing a fixed voltage magnitude and varying the pulse width according to signal information, the resulting output is represented as the capacitor node voltage according to the accumulated charge within the neuron circuit. Using a fixed voltage helps alleviate non-linear *I*-*V* characteristics in the synaptic device because the accumulated change can be linear to the pulse width of input signal. Choi et al. (2017) also utilized the pulse width modulation while keeping the input amplitude constant in the memristor CBA. To mitigate the non-linear *I*-*V* characteristics of memristor device, the input signal was encoded by varying pulse width from 0 to 1,000 μs (100 μs unit pulse width and a constant amplitude of 0.3 V) proportional to the values of the input data. Another approach to tackling non-linear *I*-*V* relationships in analog input involved PWM, where analog input was encoded into *n* pulse trains. Cai et al. (2019) implemented a multilayer neural network employing this technique. To account for the constant offset that occurs during the rising and falling times, instead of solely modifying the pulse width, they adjusted the equivalent width of the input pulse using *n* discrete-time pulse trains. Each channel was subsequently linked to a digital-to-analog circuit, which converted the 6-bit input into an n-element pulse train. Considering the *I*-*V* non-linearity, VMM operation was executed in the charge domain, and the charge accumulated in the capacitor during the application of the input pulse was utilized as the outcome of the VMM operation. Yao et al. (2020) employed shift-and-adder circuits to represent analog-grade input information, as depicted in Figure 9E. They achieved this by applying binary pulses n times, each with the same pulse width, and utilizing the shift-and-add method, starting from the most significant bit (MSB). This approach allowed them to represent 255 levels of grayscale MNIST images through 8-bit binary encoding with a 0.2 V pulse for ‘1’ and GND for ‘0’. These pulses were applied sequentially at eight-time intervals, allowing the current to flow through the shift-and-add circuitry.

#### 3.1.3 Multi-device weight implementation

Multi-device refers to representing a single synaptic weight using multiple synaptic devices (Rzeszut et al., 2022). This approach addresses the limitations posed by devices with restricted dynamic ranges and asymmetric conductance responses, which can lead to precision challenges in weight representation. Boybat et al. (2018) introduced a multi-memristive synapse architecture in which multiple PCM devices form a single synapse, as depicted in Figure 9F. This architecture resolves the issue of asymmetric weight updates caused by the abrupt reset behavior of PCM devices. Furthermore, it has been demonstrated that the conductance varies linearly with the number of devices, thereby enhancing weight resolution and facilitating more accurate weight updates. Through this strategy, temporal correlation detection was experimentally implemented using 10^3^ PCM-based synapses in a spiking neural network (SNN). As a result, this method compensates for the inherent limitations of specific synaptic devices and can potentially be applied to other synaptic device types apart from PCM. Garbin et al. (2015) also proposed the configuration of a single synapse comprising *n* binary OxRAM cells connected in parallel. The *n* devices in a row form the synaptic structure connected to the presynaptic and postsynaptic neurons. Ambrogio et al. (2018) tried to compensate for the linearity problem caused by the variation issues encountered when using analog synaptic devices. They introduced a novel 2PCM + 3T1C structure, wherein additive devices (CMOS and capacitors) were integrated into a single synapse unit. This structure aimed to achieve enhanced linearity and precise weight modulation, taking inspiration from DRAM-like operations. Their objective was to achieve weight adjustments proportional to the current flowing through the weight.

#### 3.1.4 Cell compensation

During hardware-based inference operations, performance degradation can occur due to stuck cells or resistance from other cells (Yan et al., 2017; Park et al., 2022). A single faulty cell within a memristor crossbar array can disrupt the current in the column, affecting the neuron’s output and overall yield. Two types of fault devices can exist within such arrays: soft and hard faults. Soft fault devices undergo changes in conductance due to factors like write variation, read disturbance, fabrication discrepancies, and endurance issues, leading to decreased computational accuracy. The status of these cells can be re-adjusted through tuning processes. On the other hand, hard-fault devices experience complete breakdowns, rendering their status uncontrollable. These faults may arise from process issues, resulting in the device being stuck in either LRS or HRS. LRS faults could be caused by defects during over-forming or failures during the reset switching process, while HRS faults may result from permanent electrode opening or switching failure (Xia et al., 2017; Yeo et al., 2019).

The influence of stuck cells may be more significant for offline learning, where pre-trained weights are transferred. To mitigate the impact of such stuck cells, Ma et al. (2016) investigated the utilization of redundant arrays for error correction. In addressing the issue of SA1 faults, they developed a method to detect columns where SA1 occurs and remove them during online learning. For this, they employed a larger crossbar array and implemented a technique involving the random selection and replacement of an additional column. This action was taken if a specific column accounted for more than 10% of the total training time due to the influence of SA1 and substantially reduced the mean squared error. Liu et al. (2017) addressed the impact of stuck cells through both the retraining method and redundancy cell usage. While retraining led to an enhancement in recognition rates, more substantial improvements were achieved by replacing an additional column when stuck cells were concentrated within a specific column.

#### 3.1.5 Modified weight update methods

During the key operation phase of *in-situ* training, specifically the weight update stage, the presence of device non-idealities, especially limited dynamic range, and asymmetric conductance response exerts an influence on stable and precise conductance representations. The inherent limited conductance range of the synaptic device hinders the attainment of ideal analog states *via* continuous application of infinite pulses. Essentially, subjecting the device to a sustained series of massive pulses leads to saturation of its conductance state within a constrained range. As a result, significant set pulses cause each device within a differential pair synapse structure to saturate, leading to complexities in achieving accurate representation (Nandakumar et al., 2020). Consequently, this compromises network accuracy, impacting both inference and training operations.

To address this issue, a “refresh operation” can be employed. This operation involves applying multiple reset voltage pulses to devices that have reached saturated conductance levels, thereby reducing their conductance and restoring their functionality. This modified weight update scheme can also be considered a compensation method based on signal processing, enabling precise learning by accounting for the device finite states during the weight update process. Suri et al. (2011) suggested programming schemes involving reset updates based on device state checks, along with read and write schemes, to maintain the continuous representation (retaining) of weight states in a two-layer spiking neural network array composed of two PCM cells performing LTP and LTD during operation. These approaches were demonstrated not only in PCM but also in RRAM devices (Zhou et al., 2018; Xiang et al., 2019), as well as a van der Waals hybrid synapse device-based differential pair structure (Seo et al., 2020). These findings demonstrate the feasibility of stable weight update operations through reprogrammable, gradual conductance adjustments.

In addition, synaptic devices can exhibit an asymmetric conductance response. This implies that the amount of potentiation or depression (Δ*G*) for the identical pulse varies depending on the current *G* state. This phenomenon requires more energy and time to achieve precise weight updates, leading to a degradation in training accuracy. Instable training issues can arise due to the asymmetric behavior of synaptic devices. This mismatch occurs because the direction taken by the software-based backpropagation algorithm does not align with the actual hardware device characteristics (Gokmen and Haensch, 2020). To address this, Onen et al. (2022) proposed a learning strategy, called the Tiki-Taka algorithm, by introducing an auxiliary array system. Training is conducted in the auxiliary array and the accumulated weight update (Δ*W*) is transferred to the core array, as shown in Figure 9G. Each array system includes a reference array that stores the symmetry point of the devices. By subtracting the value of this reference array, the cost term of the stochastic gradient descent (SGD) update can be eliminated. This Tiki-Taka algorithm allows synaptic devices to converge toward their symmetry point as they receive repeated pulses, compensating for the original non-ideal behavior and thereby enhancing weight convergence stability. This algorithm was experimentally demonstrated in a 2 × 2 ECRAM-based array. Research on the Tiki-Taka algorithm, which relaxes the symmetry requirements for synapse devices, is ongoing with a focus on co-design. Material improvement approaches have been pursued from a device development perspective to ensure that Δ*G* maintains a stable symmetry point even when subjected to set/reset pulses. Additionally, algorithm development efforts have aimed to enhance noise tolerance by incorporating a filtering function to refine transmitted values before transferring the accumulated gradient to the existing separate A (auxiliary) and C (core) arrays (Gokmen, 2021). Gong et al. (2022) experimentally implemented the TTv2 algorithm in hardware through open-loop training on a 12 × 4 6T1R unit cell-based RRAM array. Hardware designed to maintain a stable symmetry point and accommodate a noise-tolerant algorithm yielded superior results, achieving approximately 98% accuracy in MNIST classification, surpassing the performance of existing synapse devices and SGD-based learning accuracy.

### 3.2 SW-HW co-optimization

#### 3.2.1 Hybrid and mixed approaches

The hybrid learning method is an approach that applies two learning methods, namely *in-situ* and *ex-situ* learning, within the same network. *In-situ* training minimizes the impact of hardware defects on hidden neurons in deep networks by inherently compensating for the weights through self-adaptation (Alibart et al., 2013; Li et al., 2018). However, a drawback of *in-situ* training is the cost associated with weight updates to the hardware and additional circuits. On the other hand, *ex-situ* training is simpler in operation but vulnerable to hardware non-idealities and incurs a cost in terms of retraining since it lacks considerations regarding hardware-induced non-idealities. In the conventional *ex-situ* learning approach, if accuracy decreases due to hardware non-idealities after transferring the pre-trained weights, retraining is necessary. However, hardware non-idealities in the earlier layers can be tolerated and compensated with the adoption of the hybrid approach.

**Figure 10:**
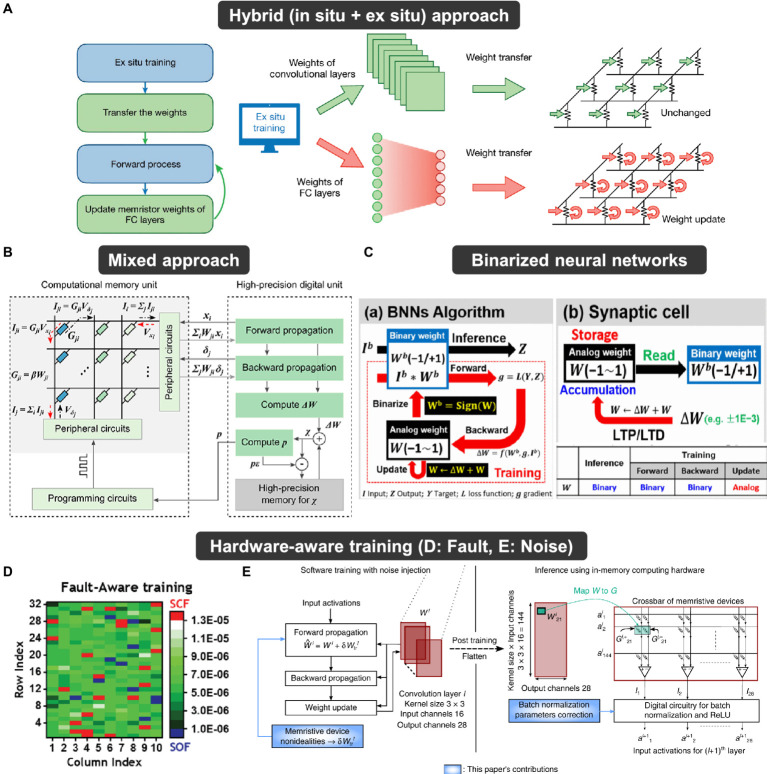
Software–hardware co-optimization approaches against non-idealities of synaptic device. **(A)** Hybrid training has eight convolution layers for *ex-situ* training and an FC layer for *in-situ* training. Reproduced with permission (Yao et al., 2020), Copyright 2020 Springer Nature. **(B)** Mixed-precision architecture consisting of high-precision processing unit and low-precision computational memory unit. Reproduced under the terms of the CC-BY Creative Commons Attribution 4.0 International License (Nandakumar et al., 2020), Copyright 2022 Frontiers Media S.A. **(C)** BNN algorithm and hardware-based BNN operations in a synaptic cell. Reproduced with permission (Zhou et al., 2018), Copyright 2018 IEEE. **(D)** Conductance map informing the locations and values of stuck cells in fault-aware training. Reproduced with permission (Yeo et al., 2019), Copyright 2019 IEEE. **(E)** Flow chart and overall methodology in variation-aware training with injecting a noise factor in the forward propagation. Reproduced under the terms of the CC-BY Creative Commons Attribution 4.0 International License (Joshi et al., 2020), Copyright 2020 Springer Nature.

Yao et al. (2020) employed a hybrid learning approach based on a memristor array for CNN, effectively addressing the challenges associated with *ex-situ* and *in-situ* training. In this approach, the convolutional layers responsible for feature extraction utilized the *ex-situ* learning method, while the FC layer responsible for the final output was trained using the *in-situ* learning method. This strategic combination compensated for performance degradation stemming from hardware non-idealities, as illustrated in Figure 10A. To assess the impact of this hybrid approach on the overall system performance, a five-layer CNN structure was implemented on a fully hardware system comprising eight packages of 128 × 16 1T1R array with TaO_x_/HfO_x_ RRAM devices, processing elements, and ARM core. For the CNN network system implementation, a 255-level grayscale image was binary encoded as input, and a total of 15 levels were employed as weights. The ideal software baseline network using 32-bit floating-point precision exhibited a CIAFR-10 recognition rate of 95.57%. However, without hybrid training and relying solely on weight transfer, a notable degradation of 79.76% occurred due to hardware non-idealities. Remarkably, following *in-situ* retraining of the FC layer, the accuracy performance demonstrated a significant improvement to 92%.

Nandakumar et al. (2020) proposed the mixed-precision computational architecture to achieve performance comparable to software-based ANN. The main operation in *in-situ* learning, the weight update phase, was addressed by proposing a new processing structure to achieve accurate and incremental conductance control and resolve precision issues in PCM devices. VMM operations and weight storage were handled by the PCM array with low precision, while the processing of VMM operation results and accumulation of weight update values were managed by a digital processing unit with high precision, as shown in Figure 10B. The experimental implementation of this mixed-precision structure demonstrated performance close to 64-bit floating-point software learning. This indicates that the hardware form of synapses, such as memristor-based crossbar arrays, can expect performance improvements by leveraging the area, energy efficiency, and parallel computation of the memristor-based arrays, along with the maturity of existing digital processing units and reduced information loss due to high precision. Kumar et al. (2022) also used the hybrid architecture based on a CMOS-based encoder unit and the emerging 2-D material h-BN-based decoder unit. They experimentally demonstrated that the mixed-precision architecture can be generalized to various edge computing tasks.

#### 3.2.2 Binarized neural networks

Binarized neural networks (BNNs), a modified type of DNN algorithm, in which weights and activations are binarized, were originally proposed to mitigate the memory access issue (Courbariaux et al., 2016). BNN employs a mixed-weight form known as binarization, allowing it to utilize analog values only during updates. Weights are binarized, except during the weight update process. When updating weights based on the accumulated gradient, real-numbered weights are utilized. These analog weights are then converted into two states through binarization. This network enables efficient hardware implementation of neural networks by adopting weight binarization to address the insufficient analog nature of synaptic devices. In HW-ANN, memory devices need to have multilevel implementation and sufficient margin between states to express precise weights. However, accurately implementing multilevel weights poses challenges due to the limited operating range (on/off ratio) of the device itself, non-linear state changes, and switching yield problems. This inaccurate representation of analog weights based on non-ideality negatively impacts accuracy and power efficiency in HW-ANN systems. By applying HW-BNN, high performance can be ensured, particularly in applications requiring high operation speed and power efficiency in arrays or large-scale neural network systems where analog weight expression is challenging due to intrinsic device non-ideality. There may be concerns about performance degradation compared to high-precision (analog) weight-based HW-ANN due to the low precision weights in BNNs. However, the BNN algorithm, proven in the deep learning community, exhibits robustness against binarization noise since the noise is averaged out through numerous multiplication and weighted sum operations, converging to the desired ideal weight. HW-BNN performance can be efficiently maintained even with hardware exhibiting non-ideal operation due to the algorithm’s robustness. From a system perspective, there are advantages in terms of computational efficiency and system energy, as matrix multiplication operations are possible with a simple XNOR operation.

Yu et al. (2016) implemented a two-layer (400 × 100 × 10) BNN on a 512 × 1,024 RRAM array in a 16 Mb RRAM macro chip. Even though the weight of the synaptic device was applied in a binary state, a high learning accuracy of 96.5% was obtained for the MNIST dataset classification task. Zhou et al. (2018) presented a hardware-based BNN demonstrating the feasibility of mitigating the degradation in on-chip learning performance attributed to the non-linearity of synaptic devices. They proposed a 2T2R cell structure for performing weight updates based on identical pulse programming, utilizing a simple peripheral circuit, where the two requirements (binary weight reading and analog weight updating) are implemented as shown in Figure 10C. Achieving precise weight updates using identical pulses is challenging due to the non-linearity in RRAM devices, which can be effectively addressed by proposed hardware-based BNNs. The accumulated change in analog weights (Δ*W*) in HW-BNN was realized as accumulated pulses applied to the devices. The binary reading stage, responsible for determining weight values as +1/−1, was achieved by comparing the conductance of the two devices (*G^+^*, *G*^−^) within a single cell as *W* = sign(*G*^+^ –*G*^−^) implemented with comparators. Even with increasing non-linearity, the accuracy of the five-layer ANN for MNIST classification remained almost constant at 97.4%. Zhang Y. et al. (2019) proposed an improved hardware-based BNN by separating the weight updating and propagation (forward and backward) modules in a 1T1R RRAM crossbar array structure, representing *W* = *G*^+^–*G*^−^, and only two states (LRS and HRS) are needed for one cell. This approach addresses two main issues in previous 2T2R cell structure-based BNN: the speed bottleneck caused by row-by-row computing during forward pass and the challenges associated with analog computations during backward pass. In addition, they successfully mitigated the impact of cell-to-cell and device-to-device variations in RRAM devices. By limiting the current during the set operation of the 1T1R cell with a lower gate voltage, the influence of LRS variation was reduced, and the propagation efficiency was increased, thereby further improving the HW-BNN. Kingra et al. (2020) demonstrated hardware-based BNN with the fabricated 8 × 8 OxRAM passive array, wherein the resistance state distribution and VMM results. To achieve analog inputs by using PWM encoding, a BNN-based ADALINE classifier was used to mitigate non-idealities by comparing the experimental (67.54%) and simulation accuracy (78.07%) classification accuracy results for the UCI Cancer dataset. This approach addressed challenges arising from imprecise state control due to the wide LRS and HRS distribution across the device. This variation was identified through measurement data, and the utilization of binary states limited the impact of variability on VMM accuracy. These approaches can be regarded as a co-design involving both hardware characteristics and algorithmic considerations beyond the synaptic devices (Qin et al., 2020; Jung et al., 2022).

#### 3.2.3 Hardware-aware training

Hardware-aware training is a learning approach where the operational characteristics of synaptic devices and arrays are considered, and then the non-idealities are identified to minimize their impact on the overall system performance in advance. This approach can be divided into two stages: (1) Detection stage: non-ideal elements of the hardware are identified and characterized. This involves detecting the conductance values, locations, and other relevant information of the non-ideal devices. (2) Tolerant training stage: once the non-ideal devices are detected, the training process incorporates the data obtained from the detection stage to train the system in a way that tolerates or compensates for the effects of the non-idealities. By following this hardware-aware training process, the neural network can adapt and optimize its performance even in the presence of non-ideal characteristics in the synaptic devices.

The effect of stuck cells, which cannot change conductance at a specific value, on system performance was reported by Bayat et al. (2018). These defects were experimentally identified through tuning. By incorporating this information into the software-based learning, the hardware-aware *ex-situ* training obtained pre-trained weights, making it aware of and importing the data from these cells. Through both simulation and experimentation methods, the effect of the hardware-aware approach was compared to the hardware-oblivious approach, where no information about these cells was considered during *ex-situ* training. They experimentally implemented a two-layer neural network on a PCB board, employing a 20 × 20 Al_2_O_3_/TiO_x_-based RRAM passive crossbar array. Both approaches were executed in 16 × 10 × 4 multilayer perceptron networks for 4 × 4 alphabet image classification. In comparison to the approach that disregarded stuck cells (with an accuracy of 79.06%), the method that considered stuck cells during training (achieving 81.4% accuracy) exhibited a better performance, demonstrating that the hardware-aware *ex-situ* training approach achieved higher classification accuracy compared to the hardware-oblivious approach. Liu et al. (2017) reported that a compensation method called the rescuing methodology, with weight significance evaluation stage, was proposed for addressing defects, specifically stuck cells, in memristor CBA. They conducted simulations based on measured data to develop this methodology. They used a 64 × 64 TaO_x_ RRAM array to read data and obtain measurements related to stuck on/off cells. By measuring the worst-case conductance data of the stuck cell defects, they determined the normal operational range of stuck on/off conductance. Using the experimental data collected from the array, the proposed retraining approach was validated through simulations. The results showed that, when applied, the retraining significantly enhanced the robustness of a two-layer neural network model for MNIST digit recognition. The accuracy of the neural network improved from 42.5 to 98.1% on average when retraining. Yeo et al. (2019) proposed the fault-aware training algorithm with experimental demonstration. The first stage involves identifying the locations and values of stuck cells within the array, as shown by the conductance map in Figure 10D. They achieve this by applying two read voltages to a cell and calculating the current–voltage ratio at those points. The current–voltage ratio of stuck cells exhibits linear characteristics compared to non-stuck cells. This detection stage helps reduce the cost of retraining. In the second stage, cells detected as stuck ones are excluded from weight updates during software training. The proposed algorithm demonstrated that even with an increased ratio of faulty cells, the recognition rate does not decrease compared to other algorithms.

Joshi et al. (2020) proposed a software-based compensation method called variation-aware training by quantifying the amount of variation in the obtained weights from reading PCM arrays. When examining the programming results for 10^4^ PCM devices at 11 conductance levels, it was observed that there was a standard deviation for the target conductance. This signified that the exact weight transfer operation was not achieved due to read/write noise inherent in the synaptic devices. To address this, the measured noise data were incorporated into the software training during inference by adding a noise factor η (the relationship between the deviation obtained based on the device with the maximum weight value within one layer and the weight value) to the weights based on a Gaussian distribution as a flow in Figure 10E. As a result, the accuracy on *ex-situ* training, which trained with additive noise, was the highest and remained consistent even during multiple inference operations.

Wan et al. (2022) implemented a synapse array of 256 × 256 RRAM cells, 256 activation functions, and several cores composed of an analog-to-digital converter (ADC) neuron circuit on one FPGA board. To maintain inference accuracy from hardware non-ideality, three hardware-algorithm co-optimization techniques were developed. (1) Model-driven chip calibration: the weights and input data of the real model were used to find the dynamic conditions for chip optimization. (2) Noise-resilient neural network training and analog weight programming: hardware non-idealities were statistically modeled during training by using methods such as injecting noises obtained through RRAM device measurements into the training process. (3) Chip-in-the-loop progressive fine-tuning: fine-tuning was performed in the forward pass process by directly calculating the error in the chip. These algorithms that consider hardware non-ideality enabled the NeuRRAM chip to secure an accuracy very similar to that of software. They confirmed the classification accuracy of the CIFAR-10 image according to the use of the algorithm. When the chip measurement was performed, the recognition accuracy decreased compared to software; however, when the proposed algorithm was used, the recognition accuracy increased by nearly 2%, and it was confirmed that its performance was very close to that of software.

### 3.3 Comparison

Table 2 summarizes the representative approaches to compensate for the hardware non-ideal characteristics, as discussed in this section. Various non-idealities present at these levels significantly impact the performance of neuromorphic systems, showing a lower correlation compared to software-based results. Through efforts within hardware and hardware-software co-optimization methods, improvements in neuromorphic system performance have been achieved. From a broader system-level perspective, the fundamental enhancements at the device and array levels result in significant performance differences, highlighting the importance of these studies. In addition, Table 3 summarizes the compensation methods for hardware non-idealities. Each method includes adjustments and application techniques customized for specific target non-idealities. Undesirable performance degradation in neuromorphic systems can result from significant hardware non-idealities, and each technique can be applied during operational phases to address such issues.

**Table 2:** Representative approaches for network performance improvement by non-ideality compensation method.

Ref.	Compensation method		Network structure (synapse array)	Network performance (before)	Network performance (after)	SIM/EXP^a^
Yao et al. (2017)	Signal/hardware engineering	Program-and-verify	320 × 3 for face image data (128 × 8 1T1R)	85.04%	88.08%	EXP
Boybat et al. (2018)		Multi devices per synapse	784 × 250 × 10 for MNIST dataset based on 9,700 PCM devices^b^	15%	88.9%	SIM
Gokmen and Haensch (2020)		Modified weight update (Tiki-Taka algorithm)	784 × 256 × 128 × 10 for MNIST dataset	15%^c^	2%^c^	SIM/EXP
Yao et al. (2020)	SW-HW co-optimization	Hybrid training	Five-layer CNN for CIFAR 10 dataset (128 × 16 1T1R)	79.76%	92%	EXP
Bayat et al. (2018)		Hardware-aware training (defect; stuck cell)	16 × 10 × 4 for 4 letters data (20 × 20 1R)	79.06%	81.4%	EXP
Liu et al. (2017)		Fault-aware training (Retraining the chip)	784 × 10 for MNIST dataset (64 × 64 1T1R array data)	42.5%^d^	98.1%^d^	SIM
Wan et al. (2022)		Noise-resilient training Chip-in-the-loop training Model driven chip calibration	ResNet-20 CNN for CIFAR-10 dataset (256 × 256 0T1R)	25.34%^e^ 83.67%	85.99%^e^ 85.66%	SIM/EXP

^a^SIM, simulation-based demonstration; EXP, experiment-based demonstration. ^b^The experimental hardware platform is built around prototype PCM chip with 3 million devices with a four-bank inter-leaved architecture. ^c^These numerical values represent the test error (%). ^d^This is the average accuracy of iterative operations, and the simulation was conducted with assumption that the same defect ratio is applied to the array data. ^e^This accuracy improvement is based on the simulation under the network structures.

**Table 3:** Summary of compensation methods, including target non-idealities, possible issues, and operating phases where each method is applied.

	Methods	Target non-ideality	Possible issue	Operating phase
Hardware/signal engineering	Program-and-verify	Variation, stuck cells	Tuning inaccuracy	Weight import
	Input encoding schemes	Variation, *I-V* non-linearity	Inaccurate output signals	Weighted sum
	Multi-device weight representation	Limited dynamic range, asymmetry, non-linearity	Limited weight precision	Weight import
	Cell compensation	Stuck cells	Inference accuracy degradation	Weight import Weighted sum
	Modified weight update	Limited dynamic range, asymmetry	Unstable weight representation	Weight update
SW-HW co-optimization	Hybrid approach	Overall non-idealities	Each limitation of *ex-situ*/*in-situ* learning	Overall operating phases
	Mixed approach	Limited precision	Limited precision, inaccurate conductance control	Weight update
	Binarized Neural Networks	Limited dynamic range, non-linearity	Limited weight precision	Weight update Weight import Weight storage
	Hardware-aware training	Overall non-idealities	Inference degradation	SW training before the weight import (Detection stage must be precedented).

## 4 Summary and discussion

In this review, we examined the types of artificial synaptic devices corresponding to the weight components containing the core information of neuromorphic systems, along with their operational characteristics. The synapse devices not only store weight states but also transmit computation results by performing VMM operations, which are the main operations of DNN. To approach ideal performance close to SW-ANNs, synaptic devices and their array structures are expected to exhibit analog (gradual) switching characteristics. Synaptic devices can be classified as conductance-based ones or capacitor-based ones. Specifically, conductance-based devices include two-terminal memristive devices and three-terminal transistor-type devices. Furthermore, we explored the synaptic array structures and weight mapping schemes depending on device structure and operational features for VMM operations.

However, when actually implementing HW-ANNs, the performance is lower than that of the software base, and various things affect the performance of the system from the device level to the smallest. In particular, non-ideal characteristics such as those at the device and array level have a great influence on the performance of the system. Thus, we anticipate hardware to exhibit excellent reliability for the successful deployment of a robust neuromorphic system. The device and array levels constitute the fundamental building blocks of a hardware-based neuromorphic system, and the non-idealities at this level are not simply eliminated or suppressed. Instead, these inevitable, non-ideal effects stemming from device limitations are addressed. Our investigation has focused on the implementation of neuromorphic systems from a compensatory and tolerant standpoint. This entails two main approaches: hardware and signal engineering, intended to be resolved within the hardware domain, and software-hardware co-optimization, a recently active research area that seeks to identify and mitigate hardware non-idealities by leveraging software assistance.

Realistically, demanding all metrics in synaptic devices for implementing a neuromorphic system is challenging. The choice between learning methods (*in-situ*, *ex-situ* training) within a network (FCN, CNN, LSTM, etc.) of a specific size, as well as the data type (binary or analog), does not critically operate for all metrics of overall system performance. This is due to the existence of primary metrics that carry varying significance based on the major operational phase (weight update, weight import, weight storage, etc.) within a particular neural network specification. For instance, it is not a given that achieving excellent linearity solely through conductance response for analog state representation would necessarily enhance network performance. This is because if conducted with *ex-situ* training, which is inference-only training, metrics like device retention and yield related to set/reset distribution could hold greater importance than linearity. Taking these application-dependencies into account, we anticipate a more robust neuromorphic system through a hybrid approach that combines complementary signal transmission and the interplay between device specifications and the surrounding CMOS/VLSI circuitry that drives the device array environment. Looking ahead, we envision a fully hardware-based neuromorphic chip that operates with stability, achieved by co-designing with the existing analog software environment, in tandem with hardware computational and information storage capabilities.

## Author contributions

KK: Conceptualization, Funding acquisition, Investigation, Writing – original draft. MS: Conceptualization, Investigation, Writing – original draft. HH: Investigation, Writing – original draft. SH: Conceptualization, Writing – review & editing. HK: Conceptualization, Funding acquisition, Project administration, Supervision, Writing – review & editing.
